# Mechanisms of Regulation of the *CHRDL1* Gene by the TWIST2 and ADD1/SREBP1c Transcription Factors

**DOI:** 10.3390/genes14091733

**Published:** 2023-08-30

**Authors:** José J. Casasnovas-Nieves, Yacidzohara Rodríguez, Hector L. Franco, Carmen L. Cadilla

**Affiliations:** 1 Department of Biochemistry, School of Medicine, University of Puerto Rico, Medical Sciences Campus, San Juan 00936, Puerto Rico; jjcasasn@gmail.com (J.J.C.-N.); yacid.rodriguez@gmail.com (Y.R.); hfranco@med.unc.edu (H.L.F.); 2 Department of Genetics, School of Medicine, University of North Carolina Chapel Hill, Chapel Hill, NC 27599, USA

**Keywords:** Setleis syndrome, TWIST transcription factors, bHLH, SREBP1c, ADD1, CHRDL1, BMP signaling

## Abstract

Setleis syndrome (SS) is a rare focal facial dermal dysplasia caused by recessive mutations in the basic helix-loop-helix (bHLH) transcription factor, TWIST2. Expression microarray analysis showed that the chordin-like 1 (*CHRDL1*) gene is up-regulated in dermal fibroblasts from three SS patients with the Q119X TWIST2 mutation. METHODS: Putative TWIST binding sites were found in the upstream region of the *CHRDL1* gene and examined by electrophoretic mobility shift (EMSA) and reporter gene assays. RESULTS: EMSAs showed specific binding of TWIST1 and TWIST2 homodimers, as well as heterodimers with E12, to the more distal E-boxes. An adjoining E-box was bound by ADD1/SREBP1c. EMSA analysis suggested that TWIST2 and ADD1/SREBP1c could compete for binding. Luciferase (*luc*) reporter assays revealed that the *CHRDL1* gene upstream region drives its expression and ADD1/SREBP1c increased it 2.6 times over basal levels. TWIST2, but not the TWIST2-Q119X mutant, blocked activation by ADD1/SREBP1c, but overexpression of TWIST2-Q119X increased *luc* gene expression. In addition, EMSA competition assays showed that TWIST2, but not TWIST1, competes with ADD1/SREBP1c for DNA binding to the same site. CONCLUSIONS: Formation of an inactive complex between the TWIST2 Q119X and Q65X mutant proteins and ADD1/SREBP1c may prevent repressor binding and allow the binding of other regulators to activate *CHRDL1* gene expression.

## 1. Introduction

Setleis syndrome (SS, MIM 227260) is a rare autosomal recessive disorder characterized by patients having scar-like depressions at each side of the forehead, eyebrows that go vertically upward, the absence of eyelashes on either eyelids or multiple rows of eyelashes in the upper eyelid and none in the lower eyelid. Additional facial features present in Setleis syndrome patients can be wrinkled facial skin, a bulbous nose, thick protruding lips and chin defects. Some cases show mild psychomotor retardation, even though their growth and development are normal. The syndrome was first described in 1963 in Puerto Rican patients from San Sebastian and Aguadilla [1], and it has been found in patients from other parts of the world, such as Spain, Germany, Japan, New Zealand and Oman [2,3,4,5,6].

The Setleis syndrome gene was mapped to chromosome 2q37.3 through a genome scan of two large Puerto Rican families and one family from Oman [7]. Two nonsense mutations were identified in the first exon of the *TWIST2* gene, which codes for a transcription factor of the basic-helix-loop-helix (bHLH) protein superfamily that has important roles in the development and differentiation of mesodermal and ectodermal tissues, especially in the craniofacial region [8]. Puerto Rican SS patients harbored a mutation in Gln 119 (Q119X) while the Omani family had a mutation in Gln 65 (Q65X) [7]. The Q119X mutation eliminates the *C*-Terminal region, which contains an important domain called the TWIST box, first shown to inhibit the transactivating activity of the Runx2 transcription factor [9]. The Q65X mutation truncates the coded protein just before the bHLH domain.

Additional TWIST2 mutations have been described in other Setleis syndrome patients. Mexican-Nahua sibs with facial and ophthalmologic features of FFDD type III were evaluated and found to have novel TWIST2 frameshift mutation, c.168delC (p.S57AfsX45), which caused a partial phenotype in both parents and two heterozygous sib [10]. Homozygosity for a TWIST2 missense mutation, c.326T>C (p.Leu109Pro), was identified in Turkish patients [11]. Indian patients with Setleis syndrome harbored homozygous deletions of a single nucleotide (c.91delC) in the TWIST2 gene, leading to the premature truncation of the protein product (p.R31GfsX71) [12]. Weaver et al. [13] reported on unrelated individuals, one with an unclassified FFDD and the other three with classic Setleis syndrome. Chromosomal microarray analyses revealed unique copy number variants of 1p36 in two individuals with duplications at 1p36.22p36.21 and one with a triplication at 1p36.22p36.21, hence providing evidence of locus heterogeneity for Setleis syndrome [14].

Two rare autosomal dominant syndromes have been associated with TWIST2 mutations. Ablepharon-macrostomia (AMS) and Barber Say syndrome (BSS) patients exhibit autosomal dominant inheritance and a more severe facial phenotype than Setleis syndrome (SS) patients. Marchegiani et al. [15] identified heterozygosity for a missense mutation in the TWIST2 gene (E75K) in ten patients with AMS from seven unrelated families, as well as heterozygosity for missense mutations (E75Q and E75A) and a 6-bp duplication at codons 77 and 78 in the TWIST2 gene, in eleven patients from nine unrelated families with BSS [15]. All four mutations in AMS and BSS impacted a very conserved glutamate residue (E75) in the basic domain.

Twist2 was first identified from a yeast-two-hybrid screen using E12 as bait and was named Dermo-1 for its expression pattern in the dermis of mouse embryos [8,16]. It was later renamed Twist2 based on its high homology to the Drosophila Twist protein and an overlapping expression pattern with another small bHLH transcription factor, Twist1. As seen with Twist1, Twist2 was found to inhibit both myogenic and osteoblast maturation [17]. During mammalian embryogenesis, Twist2 is expressed in mesodermal tissues, although temporally expressed after Twist1. The Twist2 knockout (KO) mice (in the 129/Sv background) had relatively normal embryonic development and no notable bone abnormalities but died 2–3 days after birth due to cachexia, failure to thrive and high levels of pro-inflammatory cytokines [16]. However, in a 129/C57 mixed background, the Twist2 knockout survived to adulthood showing features like those found in Setleis syndrome patients [7,16].

Several rare disorders have been shown to be caused by mutations in *TWIST1*: Saethre-Chotzen (SCS) [18], Robinow-Sorauf (RSS) [19] and Sweeney-Cox (SwCS) syndromes [20] and Craniosynostosis-1 (CRS1) [21], all presenting with craniosynostosis. TWIST1 may affect the transcription of fibroblast growth factor receptors, a gene family implicated in craniosynostosis [22]. There is significant phenotypic overlap between Sweeney-Cox syndrome, AMS and BSS, as well as similar genetic characteristics (autosomal dominant) and involvement of an extremely conserved glutamate (TWIST2 E75 and E117 in TWIST1), which deserves further study to better understand TWIST protein action [23].

One of the major characteristics of cachexia is severe wasting of body tissue that can be attributed to deregulation of energy homeostasis possibly due to high levels of pro-inflammatory cytokines [16]. This finding in the KO mice suggested that TWIST2 has a role in energy homeostasis, possibly through cytokine regulation. Interestingly, TWIST1 and TWIST2 were characterized as critical regulators of energy homeostasis in adipose tissue [24,25]. TWIST2 was shown to act as an inhibitor of the transcription factor adipocyte determination and differentiation dependent factor 1 (ADD1)/sterol regulatory element binding protein isoform (SREBP1c), frequently known as ADD1/SREBP1c, which is involved in adipocyte differentiation as well as the regulation of the LDL receptor gene, fatty acid and sterol biosynthesis genes [26]. We will refer to ADD1/SREBP1c as SREBP1c for the sake of brevity throughout the manuscript.

TWIST2 binds to consensus E-boxes in target genes as a homodimer or heterodimer with E-proteins, to function as a repressor or as an activator [27]. The Q119X mutant form of TWIST2 forms homo- and heterodimers with E12 and TWIST1, and binds E-boxes in the human Periostin (*POSTN*) gene promoter [28]. Expression profiling of RNAs derived from Puerto Rican Setleis syndrome patient dermal fibroblasts revealed a long list of differentially regulated genes, from which the chordin-like 1 (*CHRDL1*) gene stands out as having the highest positive fold-change, being up-regulated 74-fold [29]. This finding suggests that the truncated Q119X TWIST2 protein is a negative regulator of *CHRDL1* gene expression.

The *CHRDL1* gene codes for a secreted protein contain cysteine-rich repeats that act as antagonists of the bone morphogenic proteins (BMP) 2 and 4. [30,31]. BMP proteins are involved in the induction and patterning of the ventral mesoderm during embryonic development [32]. BMP signaling has an important role in the development of craniofacial structures and their function is tightly determined by temporal and spatial regulation by environmental cues [33]. The expression patterns observed for BMPs is usually opposed to that of the BMP antagonists as these signals are expressed in concentration gradients that determine the fate of cell populations at a given time [34].

Mutations in the *CHRDL1* gene cause an X-linked genetic disorder called Megalocornea (MGC1, MIM # 309300), which is an ocular anterior segment disorder caused by failure of normal development of the anterior segment of the eye [35]. Affected individuals are characterized by having an increased diameter of their corneas with no increase in intraocular pressure at birth, and can develop corneal degeneration, lens dislocation and cataracts [36]. Davidson et al. [36] performed targeted and whole exome sequencing (WES) and identified a novel missense mutation in *CHRDL1* in a male patient diagnosed with megalocornea-mental retardation (MMR) syndrome that accounts for his MGC1 phenotype, but not his non-ocular features. The authors suggested that MMR syndrome, in some cases, may be di- or multigenic [36]. Male *Chrdl1* KO mice do not have larger cornea diameters than wild type mice, but have significantly thicker central corneas and smaller anterior chamber depth [37], which is the opposite of what occurs in humans who have *CHRDL1* mutations. Therefore, the *Chrdl1* KO mice described did not recapitulate the human MGC1 phenotype.

The finding that *CHRDL1* is up-regulated in SS patient dermal fibroblasts suggests that the TWIST2-Q119X mutation found in Puerto Rican patients causes a loss of function of TWIST2 that may prevent its repression of *CHRDL1* gene expression in these types of cells. Twist2 is required for normal corneal keratocyte proliferation and eyelid morphogenesis in the developing eye of the mouse, since loss of Twist2 function reduced stromal keratocyte proliferation and caused corneal thinning [38]. In humans when the *CHRDL1* gene is defective, corneas are thin and of increased diameter. *Twist2* knockout mice have thin central corneas, while the *Chrdl1* KO mice have thicker central corneas. If Twist2 represses *Chrdl1* gene expression, when Twist2 is absent, one would expect to have higher *Chrdl1* gene expression, which presumably should have the opposite effect in corneas than that seen in *Chrdl1* KO mice, i.e., corneal thinning rather than corneal thickening, which is what is observed in these mice.

TWIST2 has been shown to act as a physical inhibitor of the transcription factor SREBP1c by interacting with the N-terminus of SREBP1c [26]. This bHLH transcription factor is involved in adipocyte differentiation as well as regulation of the LDL receptor gene, as well as fatty acid and sterol biosynthesis genes [39]. In humans, there are three major SREBP isoforms: SREBP-1a, SREBP-1c, and SREBP2 [40,41]. SREBP-1a and SREBP-1c, which are generated by alternative promoter usage and alternative splicing from a single gene, and are involved in the regulation of fatty acid, phospholipid, and triacylglycerol synthesis [41]. SREBP1 is expressed membrane-bound to the endoplasmic reticulum and upon activation, proteases cleave the cytoplasmic part of the protein, causing the transcription factor to migrate into the nucleus. On the other hand, SREBP2 is encoded by a separate gene and is mainly involved in controlling the expression of genes involved in the uptake and biosynthesis of cholesterol [39,40,41]. When compared with SREBP1a and SREBP2, SREBP1c is a weaker transcription activator. This is mostly due to the few acidic amino acids in its *N*-terminal region compared with SREBP1a and SREBP2 [39,40]. *CHRDL1* gene expression was up-regulated during adipogenesis in primary cultured and established mesenchymal progenitor cell lines [42] and this up-regulation promoted adipocyte differentiation. Our hypothesis is that the Q119X *TWIST2* mutant protein fails to repress the *CHRDL1* gene in human fibroblasts, which leads to its overexpression. The *CHRDL1* 5′-flanking region has yet to be rigorously characterized to understand its involvement in eye and facial development, adipocyte differentiation and its implications in Setleis syndrome.

In a previous study, we examined the evolutionary relatedness of the Twist1 and Twist2 proteins and identified a highly conserved sequence motif that is found in the majority of Twist1 and Twist2 vertebrate proteins, particularly amongst the mammalian class where they are strictly conserved [43]. In this work, we examined regulatory mechanisms of the human *CHRDL1* gene by the TWIST2 and SREBP1c transcription factors. In order to better understand how TWIST2 may regulate *CHRDL1* gene expression, we studied the human *CHRDL1* 5′-flanking region and identified putative E-boxes that may be recognized by Twist proteins and performed DNA binding and reporter gene assays to determine if TWIST2 represses the human *CHRDL1* gene and compared the DNA binding capacity of wild type and mutant forms of TWIST2 protein to the regulatory region of the *CHRDL1* gene.

## 2. Materials and Methods

### 2.1. Bioinformatics Analysis of Putative Binding Sites (E-Boxes) in the Human CHRDL1 Gene Upstream Sequence 

Analysis of the regulatory region (spanning ca. 3000 bp upstream from the transcription start site) of the *CHRDL1* gene was carried out using the web-based tool Transcription Element Search System (TESS) (http://www.cbil.upenn.edu/cgi-bin/tess/tess, accessed on 23 February 2007, this service is no longer available) [44]. Multiple sequence alignments comparing the regulatory region of CHRDL1 from different mammals were carried out using the mVISTA web tool (http://genome.lbl.gov/vista/index.shtml, accessed on 9 May 2023) [45,46]. The reference sequence from the human genome database at the NCBI website (http://www.ncbi.nlm.nih.gov/gene/, accessed on 9 May 2023) for the regulatory region of the human *CHRDL1* gene was submitted to TESS and mVISTA analysis using the default settings and parameters.

### 2.2. Cell Culture 

Normal skin fibroblasts (GM00637) were cultured using Minimum Essential Medium (MEM) Eagle with Earle’s salts supplemented with 10% fetal bovine serum (FBS) 1% penicillin/streptomycin. HeLa cells were cultured and maintained in Dulbecco’s modified Eagle’s medium (DMEM) supplemented with 10% fetal bovine serum (FBS), 1% L-glutamine, and 1% penicillin/streptomycin. COS-7 cells and HEK293T cells were cultured and maintained in DMEM supplemented with 10% fetal bovine serum (FBS), 1% L-glutamine, 1% penicillin/streptomycin, 1% HEPES, and 1% sodium pyruvate. All cell lines were cultured at 37 °C with 5% CO_2_ in a Tissue culture incubator (Thermo Scientific, Waltham, MA, USA).

### 2.3. Plasmid Constructs and Site-Directed Mutagenesis

Mutagenesis reactions to delete the first conserved sub-motif SSSPVSP (TWIST2ΔA) or the acidic second conserved sub-motif SEEE (TWIST2ΔB) were performed using the QuikChange II Site-directed mutagenesis Kit (Agilent, Santa Clara, CA, USA) as recommended by the manufacturer. A plasmid construct containing the myc-tagged cDNA insert coding for human wild-type TWIST2 in the pCDNA 3.1 (+) vector was used as template for site-directed mutagenesis (donated by Drs. Drazen Sosic and Eric Olson, UT Southwestern). Primers containing the desired mutation were designed according to the mutagenic primer design guidelines and web-based primer design software program (http://labtools.stratagene.com/QC, accessed on 9 May 2023) provided by the manufacturer, as described in the manufacturer’s protocol. For the mutant TWIST2ΔAB, deletion of both N-terminal sub-motifs was performed via sequential mutagenesis. Once the mutant TWIST2ΔA construct was created and confirmed by Sanger sequencing, the pcDNA3.1 TWIST2ΔA plasmid construct was used as a DNA template for deletion mutagenesis of the second sub-motif using the primers used for the TWIST2ΔB mutation. The primer sequences for generating the deletion constructs can be found in Supplementary Table S1. Sanger Sequencing was carried out by the UPR Medical Sciences Campus RCMI Molecular Biology Core facility for confirmation of the desired mutations using the T7 Forward primer (5′-TAATACGAC TCACTATAGGG-3′), and the BGH Rv primer (5′-CTAGTTATTGCTCAGCGGTG-3′) in both strands. Supplementary Figure S1A shows a diagram of the domains found in the TWIST2 deletion constructs, compared to the TWIST2 wild-type protein. Supplementary Figure S1B shows the domain structure of the TWIST1 wild-type protein and the deletion constructs we used in this study. Myc-tagged TWIST1 deletion constructs in the pcDNA3.1(+) vector (Twist1ΔGly1, Twist1ΔGly2, Twist1ΔGly1&2 and Twist1_N-terminus (T108X)) were prepared by gene synthesis, by a commercial provider (Gene Art Synthesis, Life Technologies) using their online portal and following their online portal tutorial for construct design (www.lifetechnologies.com/genesynthesis, accessed on 8 March 2017). Plasmids constructs in the pCDNA 3.1 (+) vector containing myc-tagged cDNA inserts coding for human TWIST1, wild-type TWIST2, mutant forms of TWIST2 (Q119X and Q65X) [7] as well as a construct containing the coding region for E12 cloned in the pCITE vector (Millipore Sigma) were a gift from Dr. Drazen Sosic and Dr. Eric Olson, UT Southwestern. A construct containing the coding region of the TCF3 variant E12 was cloned by T/A cloning in the pCRII-TOPO vector (Invitrogen) and referred to as the TCF3 construct (see PCR primer sequences in Supplementary Table S1). The RNA used for the reverse transcriptase-PCR reaction was obtained from human GM00637 SV-40 transformed dermal fibroblasts. This TCF3 construct had an upstream T7 promoter, which was used for producing E12 in vitro. The coding sequence for amino acids 1–403 of rat SREBP1c cloned in the pcDNA3.1/myc-His A vector was a gift from Dr. Jae Bum Kim (Seoul National University [26]).

### 2.4. In Vitro Protein Synthesis, and Electrophoretic Mobility Shift Assay (EMSA) 

In vitro translated proteins were produced using the TnT Quick Coupled Transcription/Translation system (Promega) with 1 μg of template plasmid DNA as recommended by the manufacturer. For production of every individual protein or heterodimers, two reactions were sets of 10 μL and 40 μL. All reactions were incubated at 30 °C for 1 h. The 10 μL reaction was labeled with 5 μCi of ^35^S-methionine (1000 Ci/mmol) and denatured at 80 °C for 15 min in SDS loading buffer containing 10% β-mercaptoethanol. Samples were run in a 10% SDS-PAGE, after which gels were soaked for 30 min in fixing solution (50% methanol, 10% glacial acetic acid, 40% water) followed by soaking in 10% glycerol for 30 min. The gel was dried in a BIO-RAD gel dryer at 80 °C for at least 4 h, followed by at least 4 h at room temperature. Dried gels were exposed on X-ray films for at least 16 h at room temperature. The 40 μL reaction did not contain radiolabeled methionine, hence, 0.8 nanomoles of methionine were added instead of the ^35^S-methionine. For the production of heterodimers, 1 μg of each of the two plasmids (coding for TWIST2 and E12 proteins) were added for a total of 2 μg of plasmid DNA. Binding reactions for Electrophoretic Mobility Shift Assays (EMSAs)were carried out in binding buffer (20 mM HEPES pH 7.9, 60 mM KCl, 1 mM MgCl_2_ 0.5 mM dithiothreitol and 10% glycerol) using 1–3 μL of the in vitro transcription/translation reactions or 1–2 μg HeLa nuclear extracts, in a final reaction volume of 10 μL. Probes used for EMSAs were either 30–40 bp long double strand oligonucleotides or PCR products, which were end-labeled with ^32^P using T4 polynucleotide kinase (New England Biolabs) and 10 μCi of γ-^32^PATP (Amersham). Reaction products were purified using spin columns (Illustra Microspin G-25 Column, GE Healthcare) following manufacturer’s instructions. Competition reactions for EMSA were set up using a molar excess (at least 50 times the molar concentration of the labeled probe) of unlabeled specific and/or non-specific oligonucleotides (an oligonucleotide containing a GC box bound by the SP1 transcription factor). For supershifts, 200 ng of antibody (anti-Myc antibody, Santa Cruz, sc-40) were used in the binding reactions, which were loaded on a 6% polyacrylamide (29:1) native gel containing 2.5% glycerol and 0.5X TBE as gel and running buffer previously pre-run for 2 h at 100 V constant voltage, at 4 °C in a cold room. Gels were run for at least 5 h at 4 °C at a rate of 5.5–6 V/cm (constant voltage) and dried on a BIO-RAD gel dryer applying vacuum and heat (80 °C) for 1 h and exposed to an X-ray film or phosphor-imager screen (BIO-RAD Molecular Imager GS-525) for at least 18 h at −80 °C.

### 2.5. Luciferase Reporter Gene Assay 

A luciferase reporter gene construct was prepared by amplifying a 2836 bp fragment of the *CHRDL1* regulatory region through PCR. This fragment contained 5 E-boxes and was directionally cloned into the KpnI and XhoI sites of the pGL4.14 luciferase reporter vector (Promega) polylinker region and named -3K*CHRDL1*-pGL4. The forward primer (CH3KLuc-FW) contained additional bases at the 5′, which added a KpnI restriction site, and the reverse primer (CH3KLuc-RV) added a XhoI restriction site at the end of the amplicon (See Supplementary Table S1 for primer sequences). The Advantage 2 Polymerase mix (Clontech) was used for amplification. The PCR cycling program used was: 94 °C for 1 min for the initial denaturation and polymerase activation, 40 cycles composed of: 94 °C for 30 s, 65 °C for 30 s and 72 °C for 3 min, and finally 72 °C for 5 min after the cycling. Reaction products were cloned in the pCR^®^II vector using the TA cloning system (Invitrogen). The cloned fragments were excised from the pCR^®^II plasmid by digestion with Xho I and Kpn I, purified by gel extraction from a 0.8% (*w*/*v*) agarose gel and directionally cloned into the pGL4.14 luciferase reporter vector (Promega) previously cut with XhoI and KpnI. The ends of the insert of this construct were verified by Sanger sequencing at the UPR MSC RCMI Molecular Biology Core using dye terminator sequencing chemistry.

Human SV-40 transformed skin fibroblast GM00637 cells (2 × 10^5^) in 6-well plates were co-transfected with 1.5 μg of the -3K*CHRDL1*-pGL4 reporter gene and 100 ng of the pGL4.75 vector, which expresses Renilla luciferase driven by a CMV promoter (as an internal control for normalization) using the Fugene HD transfection reagent (Roche) at a 4:2 ratio of μL of transfection reagent to microgram of DNA. In addition, the cells were co-transfected with 250 ng of mammalian expression constructs described above containing Myc-tagged TWIST1, TWIST2 in its wild-type and mutant forms, and/or myc and His-tagged ratADD1/SREBP1c encoding amino acids 1–403. Transfected cells were incubated for approximately 48 h prior to active lysis after scraping them from the culture dish using the Reporter Lysis Buffer as recommended by the manufacturer (Promega) and then transferred to a microcentrifuge tube. These extracts were then submitted to three (3) freeze–thaw cycles using a dry ice-ethanol bath. Luciferase assays were carried out using the Dual Luciferase Reporter Assay Systems kit (Promega). Luciferase activity was measured in an MLX microtiter plate luminometer (Dynex technologies). The firefly/Renilla ratio obtained for the samples transfected with the pGL4.14 empty vector was normalized to 1 and comparison of the different samples was carried out against this sample. Statistical analysis of the luciferase results was performed using Student’s *T*-test.

The -3K*CHRDL1*-pGL4 reporter gene construct was also transfected into about 1 × 10^4^ or 2 × 10^5^ HeLa or COS-7 cells, respectively, which were seeded in six-well plates and grown in their respective media for 24–48 h to reach 70–90% confluence. HeLa cells were transiently transfected or co-transfected using the Lipofectamine 3000 reagent (Invitrogen) with 4.9 μg of plasmid DNAs from mammalian expression vectors for ADD1/SREBP1c, WT TWIST2, TWIST2ΔA, TWIST2ΔB, TWIST2ΔAB, TWIST2-Q119X, TWIST2-Q65X, WT TWIST1 or pGL4.14 Empty vector, and pGL4 Promoter vector), and 100 ng of pGL4.75 (Promega). Prior to transfection, the DNA was diluted in 125 μL of serum-free Opti-MEM Medium (Life Technologies), and 10 μL of the P3000 reagent (an enhancer reagent that is supposed to increase stabilization of the lipid/DNA complexes) was then added to the diluted DNA and mixed well. Then, 5 μL of the Lipofectamine reagent was diluted in 125 μL Opti-MEM medium and mixed well. The diluted DNAs were then added to each tube of the diluted Lipofectamine at a 1:1 ratio and incubated for at least 15 min at room temperature to allow formation of lipid–DNA complexes. During incubation, cell culture media were removed and replaced with 2 mL of Opti-MEM Medium. When incubation time was over, 250 μL of the transfection medium (DNA-lipid complexes) was added drop by drop to the cells to avoid disruption of the complexes. Cells were then incubated at 37 °C with 5% CO_2_. At 8 h post transfection the transfection medium was removed and replaced with 2 mL of fresh new DMEM medium. The cells were incubated for an additional 48 h prior to passive lysis.

For COS-7 cells, cells were transiently transfected or co-transfected with 40 ng of pcDNA3.1 construct, 240 ng of PGL4 Empty vector, 240 ng -3K*CHRDL1*-pGL4 reporter gene vector, and 16ng of pGL4.75. Prior to transfection, 4 μL of lipofectamine 3000 was diluted in 250 μL of Opti-MEM medium and mixed well, and the DNA was diluted in 250 μL of Opti-MEM medium followed by the addition of 1 μL of the P3000 reagent and mixed well. The diluted DNAs were then added to each tube of the diluted Lipofectamine at a 1:1 ratio and incubated for at least 15 min at room temperature. Cell culture medium was removed and replaced with 2 mL of Opti-MEM Medium. When incubation time was over, 500 μL of the transfection medium (DNA-lipid complexes) were added and cells incubated at 37 °C with 5% CO_2_. At 4 h post transfection the transfection medium was removed and replaced with 2 mL of fresh new DMEM medium. The cells were incubated for 48 h prior to passive lysis.

In addition, transient transfections as described above were also conducted using the COS-7 cell line, but 24 h prior to cell lysis for Luciferase Assay, 1 mM of sodium butyrate (NaBt) was directly added to the cells in the 6-well plates to inhibit histone deacetylases (HDACs).

After 48 h of transfection of HeLa or COS-7 cells, the media were removed, and cells were washed with 2 mL 1X PBS. The 1X Passive Lysis Buffer (PLB) was prepared just before use by mixing 1 mL of 5X PLB + 4 mL of dH2O. The cells were then passively lysed with 500 μL of 1X Passive Lysis Buffer (PLB) for HeLa cells, 200 μL of PLB for COS-7 cells, and 100 μL of PLB for HEK293T cells, and the plates were covered with aluminum foil and placed on a rocking platform for 15 min to 1 h at room temperature, followed by harvesting by scraping with a disposable rubber policeman. The lysed cells were then transferred to a 1.5 mL tube and centrifuged for thirty seconds in a microcentrifuge at maximum speed at room temperature.

Luciferase Assays for HeLa or COS-7 cell transfections were carried out as recommended by the manufacturer in a single cuvette luminometer (Promega GloMax^®^ 20/20 Luminometer). The relative luciferase activity was read from the instrument, which was expressed as the ratio of firefly luciferase and Renilla luciferase. For maximal accuracy, the results were normalized from each experimental sample to promoter control sample by the ratio of experimental/control reporter activity as described [47]. Luciferase assay data were analyzed by one-way ANOVA followed by unpaired Student T-test to compare with the promoter control of the experimental group. The experiments were repeated at least three times independently (*n* = 3) or five times (*n* = 5). Data analysis was performed using Prism 4 Graph Pad Software. Differences of *p* < 0.05 were significant. Error bars represent standard error of the mean.

## 3. Results

### 3.1. Bioinformatic Analysis Reveals That the 5′ Upstream Region of the Human CHRDL1 Gene Contains Putative Transcription Factor Binding Sites Which Are Conserved in Mammals

#### 3.1.1. Transcription Element Search System (TESS) Analysis

**Figure 1:**
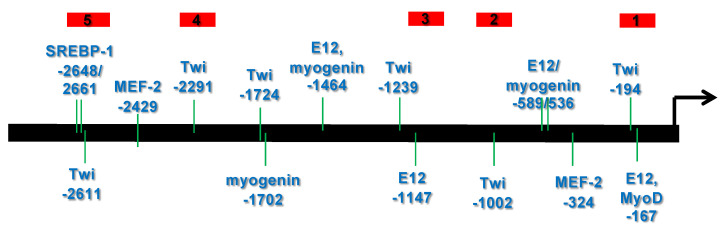
Schematic representation of the putative binding sites identified by the bioinformatic analysis using TESS. Five putative TWIST binding sites were identified in the 5′ upstream region, relative to the transcription start site of the *CHRDL1* gene. In addition, putative binding sites for other proteins known to interact with TWIST2 were also predicted by TESS. The distance between sites is not in scale, but the diagram shows the relative positions of the putative binding sites. Based on this analysis we designed five probes, shown here as red bars, for carrying out DNA binding assays by EMSA. Probe #1 comprises nucleotides from −240 to −58, probe #2 comprises from −1044 to −917, probe #3 comprises from −1297 to −1148, probe #4 comprises from −2297 to −2198, and probe #5 comprises from −2697 to −2548 (relative to the transcription start site in human reference sequence NG_012816 REGION: 2242.127203).

To determine if there were any possible TWIST2 binding sites in the *CHRDL1* gene 5′ flanking region, we performed bioinformatic analysis of the upstream sequences of the human *CHRDL1* gene. For this analysis, we employed the TESS tool available at the University of Pennsylvania website. TESS analysis rendered a list of putative binding sites of different transcription factors. We focused on those elements that we considered biologically relevant to the role of TWIST2 (Figure 1). The TESS analysis identified putative binding sites, at positions relative to the transcription start site, for E12/E47 (−1464, −589, −536, −167), MyoD (−167), myogenin (−1702, −589, −536), SREBP1 (−2661, 2648), MEF-2 (−2429, −1972, −324) and TWIST (−2611, −2241, −1239, −1002, −194). The TWIST element consensus sequence used by TESS is for the *Drosophila* TWIST protein, which Li et al. [8] showed that TWIST2 can bind oligonucleotides harboring this element. TESS analysis did not identify a putative TATA binding protein (TBP) site/TFIID site at the expected location.

#### 3.1.2. mVISTA Analysis of Mammalian Chordin-like 1 Genes

We compared the upstream regions of Chrdl1 genes from different mammalian species using the mVISTA web-based tool for multiple sequence analysis. We found that the Chrdl1 upstream regions are conserved in macaque, chimpanzee, orangutan, horse, dog, pig, squirrel, mouse, and rat (Supplementary Figure S2A). The putative binding sites for TWIST in the human *CHRDL1* gene were crossed-checked in the multiple sequence alignment and found to be conserved in most of the species compared. Interestingly, in the case of the −1239 TWIST putative binding site, the sequence conserved in macaque, chimpanzee, pig and squirrel is CACTTG, which is an E-box, but in orangutan this sequence is CGCTTG, which is a D-box (Supplementary Figure S2).

### 3.2. Several bHLH Transcription Factors Have DNA Binding Activity to Upstream Region Sequence of the Human CHRDL1 Gene

To gain insight into which proteins might be binding to probes #1 and #5 (See Figure 1 above) or oligonucleotide probes derived from these regions, we used in vitro-synthesized proteins. Similar quantities of protein were synthesized in vitro. No instability issues were observed when the proteins were analyzed by SDS-PAGE prior to carrying out EMSAs (Supplementary Figure S3A,B).

**Figure 2:**
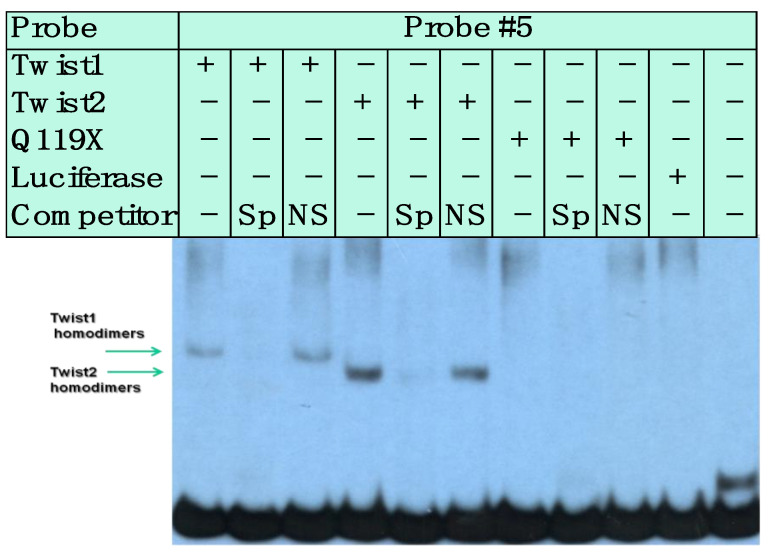

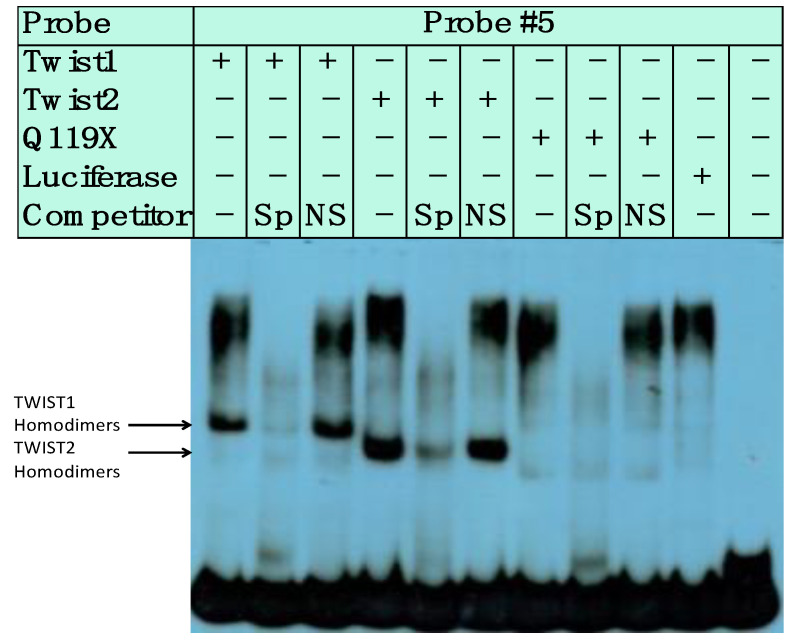
Electrophoretic mobility shift assay for the most upstream region of the *CHRDL1* gene tested with TWIST1 and TWIST2 proteins. Typical results of EMSA carried out with the in vitro synthesized TWIST proteins using probe #5. The table above the figure indicates with a plus (+) sign the proteins expressed in the TnT extracts and the unlabeled oligonucleotide competitors added to the DNA binding reactions. A minus (−) sign indicates the absence of an expressed protein or competitor oligonucleotide in the reaction. SP = specific oligonucleotide competitor; NS = non-specific oligonucleotide competitor. The (**top image**) represents a 15 h exposure, while the (**bottom image**) is from a 36 h exposure to X-ray film. Both TWIST1 and TWIST2 homodimers were able to bind this probe, but binding of the TWIST2 Q119X mutant protein could only be detected when overexposing the dried gel (**bottom image**). The binding is specific due to being competed out by an unlabeled probe but not by the unlabeled Sp1 element probe. Another shift is observed close to the wells’ origin but is considered non-specific binding as it is also observed in the luciferase lane (mock reaction).

No binding was detected for TWIST1 or TWIST2 proteins in homo- or heterodimer forms to the probe #1 region, but binding for E12 and TCF3, which are other isoforms of the E2A gene (Supplementary Figure S3C), was detected. In the case of probe #5, which contained at least three E-boxes, we detected specific binding of TWIST1 and TWIST2, since no competition was observed when a non-specific competitor was added to the binding reaction (Figure 2 Top). The shift observed for TWIST2 gave a more intense signal than the shift observed for TWIST1. We typically exposed the X-ray film for about 18 h before developing it. However, to detect possible weak interactions, the exposures were repeated leaving the X-ray film for a prolonged exposure of 36 h. Interestingly, when this EMSA was overexposed in this manner, weak binding of the Q119X mutant form of TWIST2 was observed, though this binding seems to be non-specific, since no competition was observed when a specific competitor was added (Figure 2 Bottom). 

### 3.3. SREBP1c/Binds to the Most Upstream Ebox Site along TWIST1 and Wild Type TWIST2

**Figure 3:**
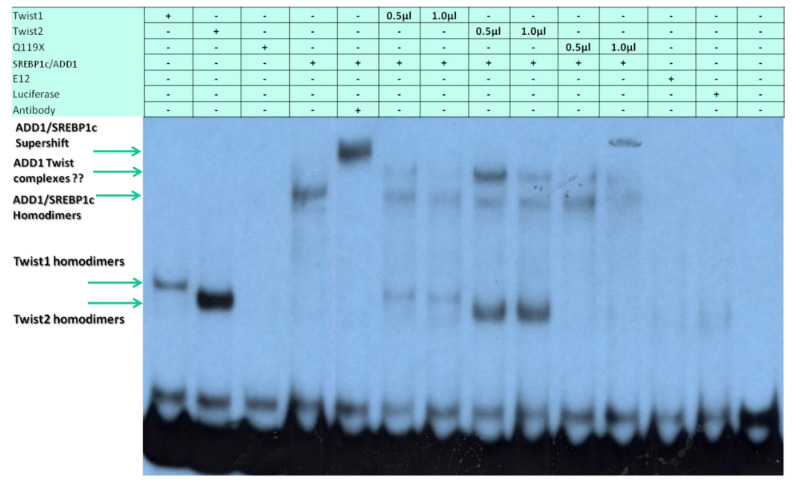
Electrophoretic Mobility Shift Assay for the most upstream region of the *CHRDL1* gene with TWIST, E12 and SREBP1c bHLH proteins. EMSA carried out with probe #5 to assess the SREBP1 putative binding site adjacent to the TWIST binding sites. The table above the gel image indicates the protein or oligonucleotide reagents added (plus (+) sign) or not included (minus (−) sign) in the DNA binding reactions. Since we observed binding of TWIST1 and TWIST2 homodimers to this probe, we assessed whether the SREBP1 putative binding site was bound by SREBP1c since this transcription factor is known to interact with TWIST2. A shift for SREBP1c was detected and confirmed by a supershift reaction accomplished using an antibody against the c-Myc tag of SREBP1c. In addition, we detected what could be intermediate complexes of SREBP1c /TWIST2, but their stoichiometry was not clear.

Based on the finding that both TWIST1 and TWIST2 were able to bind to probe #5, we decided to focus on better understanding the cis elements present in this probe. Bioinformatic analysis indicated that two putative binding sites for Sterol Regulatory Element Binding Protein 1 (SREBP1) existed at positions −2661 and −2648 (Supplementary Figure S2C) and one TWIST site at position −2611. The *SREBP1* gene codes for two isoforms were SREBP1a and SREBP1c, and the latter is also known as adipocyte determination and differentiation-dependent factor 1 (ADD1). While SREBP1a is more involved in regulating fatty acid metabolism, SREBP1c is more involved in adipocyte differentiation and has been shown to interact with TWIST2, leading to the inhibition of its transactivation activity [26]. We found that myc-tagged SREBP1c was able to bind to probe #5, which we confirmed by performing a supershift using an antibody against c-Myc (Figure 3). When TWIST1, TWIST2 or the Q119X TWIST2 mutant proteins were added together with SREBP1c, reduced amounts of TWIST1, TWIST2 or SREBP1c homodimers were detected. An additional complex was detected that migrated slower than the SREBP1c homodimers, which could represent TWIST2 interaction with the SREBP1c homodimer, forming a heterotrimeric complex, but its nature and stoichiometry needs further investigation. A similar possible heterotrimeric complex was detected in the TWIST1 + SREBP1c reaction, but it had lower intensity, which suggests that TWIST1/ SREBP1c complexes may have low DNA binding activity. The reaction where the TWIST2 Q119X mutant and SREBP1c proteins were mixed had very little SREBP1c homodimers binding to the probe, which suggests that the TWIST2 Q119X mutant protein forms a complex with SREBP1c that does not have DNA binding activity (the complex that migrates above SREBP1c homodimers was barely detectable), since doubling the amount of the TWIST2 mutant protein caused no detection of SREBP1c homodimeric complexes. We did not detect E12 binding to this region as a homodimer, as it did to the probe #1 region.

### 3.4. SREBP1c Can Bind Both the CHRDL1 Gene −2661 and −2648 E-Boxes While TWIST2 Prefers to Bind the −2648 E-Box, SREBP1c and TWIST2 Compete for Binding to These E-Boxes

**Figure 4:**
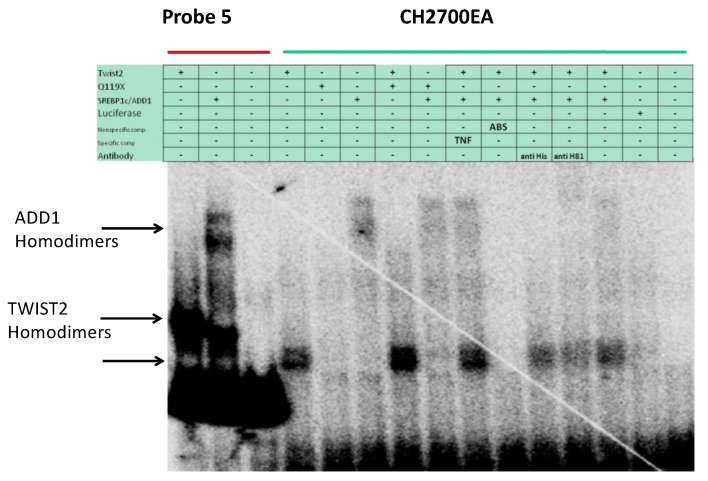
EMSA using the CH2700EA double-stranded oligonucleotide. For this probe, we detected binding of both TWIST2 and SREBP1c homodimers. The table above the gel image indicates the protein or oligonucleotide reagents added (plus (+) sign) or not included (minus (−) sign) in the DNA binding reactions. As seen before, no binding of Q119X homodimers was detected. Probe #5 was used as a positive control for the EMSA binding reactions.

**Figure 5:**
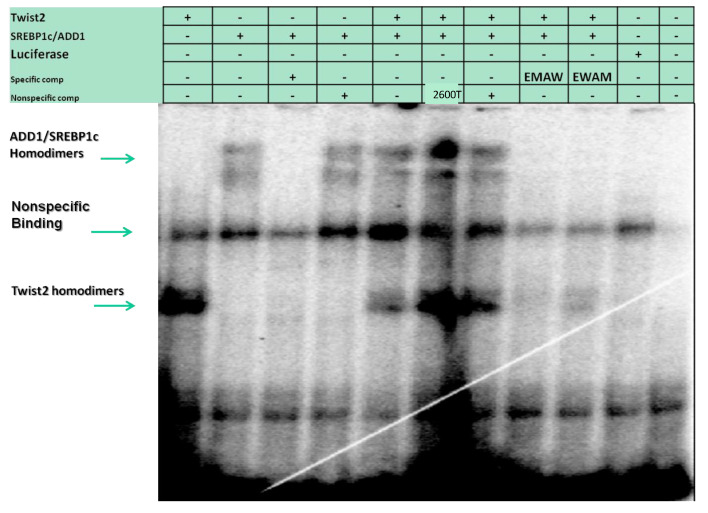
EMSA assessing the E-box preference for SREBP1c and TWIST2 using the CH2700EA oligonucleotide as probe. The table above the gel image indicates the protein or oligonucleotide reagents added (plus (+) sign) or not included (minus (−) sign) in the DNA binding reactions. Both homodimers of TWIST2 and SREBP1c were able to bind the CH2700EA oligonucleotide. In addition, we confirmed that these proteins do not bind the −2611 site since the CH2600TWI oligonucleotide did not compete out the binding of both proteins. When the CH2700EMAW oligonucleotide was used as a competitor, the shifts for SREBP1c and TWIST2 were competed out. Interestingly, when the CH2700EWAM oligonucleotide was used, it competed out the binding of SREBP1c but only competed partially for the binding of TWIST2. While SREBP1c seems to be able to bind both E-boxes, TWIST2 may bind strongly the −2648 E-box since it was not competed out completely.

**Figure 6:**
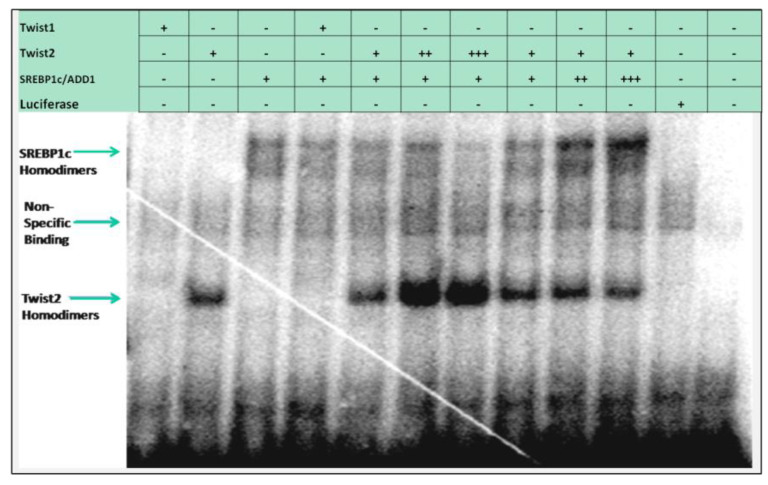
EMSA of the CH2700EA oligonucleotide with varying amounts of TWIST2 and SREBP1c. To evaluate whether TWIST2 and SREBP1c can bind simultaneously to the same region or if both proteins compete for binding, EMSAs were carried out varying the amount of these two bHLH factors. The table above the gel image indicates the protein or oligonucleotide reagents added (plus (+) sign) or not included (minus (−) sign) in the DNA binding reactions. When including increasing amounts of a given protein’s TnT reaction, two or three + signs were indicated in the table above the gel. In this EMSA, we first tested binding of both SREBP1c and TWIST2 homodimers individually. When we kept a constant amount of SREBP1c and increased the amount of TWIST2, the shift for SREBP1c was weakened. The opposite was also true; in the presence of increasing amounts of SREBP1c, the binding of TWIST2 decreased. These results suggest that both proteins compete for the same binding site. TWIST1 binding to this oligonucleotide was weakly detected.

In addition to the −2661 and the −2648 sites, probe #5 contains an additional E-box at position −2372, which was identified by TESS as a putative TWIST binding site (Figure 1). The E-box in position −2648 is conserved in human, macaque, chimpanzee, orangutan, horse and pig, while the −2661 TWIST site is conserved in human, macaque, chimpanzee, orangutan, horse, dog, and squirrel (Supplementary Figure S2C). The −2611 site is conserved in most animals considered in the mVISTA alignment except mouse and rat (Supplementary Figure S2C). To further characterize the binding preference of TWIST2 and SREBP1c between these three E-boxes, small double-strand oligonucleotides were designed to contain either the −2611 site (CH2600Twi) or the −2661 and the −2648 sites (CH2700EA) (See Supplementary Table S1). Since the CH2700EA contains two adjacent E-boxes, mutant versions of this oligonucleotide were designed in which either one of the adjacent E-boxes was ablated (CH2700EMAW and CH2700EWAM) (See Supplementary Table S1). When the CH2600Twi probe was assessed for binding of TWIST1, TWIST2, Q119X, or SREBP1c, no shift was observed. For the case of CH2700EA, a shift was observed for TWIST2 and SREBP1c, but not for Q119X (Figure 4). Next, we tested if the mutant versions of CH2700EA were able to compete for TWIST2 and SREBP1c binding (Figure 5). When the CH2700EMAW oligo was used, it was able to compete out both TWIST2 and SREBP1c, but in the case of the CH2700EWAM oligo, it was able to compete out SREBP1c and TWIST2, but a small amount of TWIST2 homodimeric binding was detected (Figure 5). Taken together, SREBP1c can bind both the −2661 and the −2648 E-boxes while TWIST2 appears to bind to the −2648 E-box strongly. Since −2661 and −2648 are six nucleotides apart and both TWIST2 and SREBP1c can bind the −2648 element, we decided to assess whether TWIST2 and SREBP1c could bind this region at the same time or if both factors compete for binding. We tested these possibilities by leaving a constant quantity of one of the proteins and increasing the other. When we increased TWIST2, we saw a decrease in SREBP1c binding, and vice versa; when SREBP1c was increased, a decrease in TWIST2 binding was observed (Figure 6).

### 3.5. The TWIST2 Q119X Mutant Protein Can Bind the Most Upstream Site of the CHRDL1 Gene as a Heterodimer with E12

**Figure 7:**
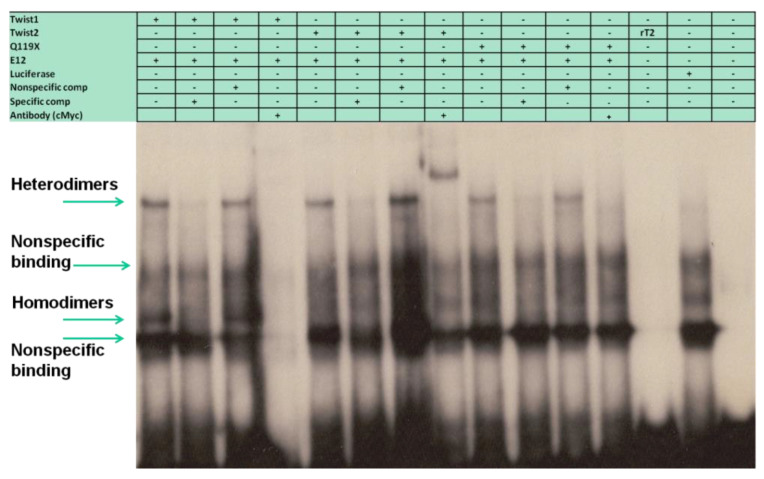
EMSA of TWIST/E12 heterodimers for evaluation of binding to probe #5. In vitro coupled transcription/translation reactions were set up using a plasmid containing the cDNA for TWIST1, TWIST2 or Q119X, combined with another plasmid containing the E12 coding sequence. The proteins obtained from these reactions were used in EMSA binding reactions containing probe #5. In this EMSA, we observed both homodimeric and heterodimeric binding. We detected binding of TWIST/E12 heterodimers, TWIST2/E12 heterodimers, and TWIST2-Q119X/E12 heterodimers. These mobility shifts were confirmed to be specific since they were competed out by the CH2700EA double stranded oligonucleotide used as a specific competitor, but the SP1 oligonucleotide did not compete. Supershift reactions confirmed that the complexes detected were from the in vitro translated proteins.

We evaluated the possibility of heterodimeric binding of TWIST1 and TWIST2 with E12 by including both expression constructs in the in vitro TNT reactions. We found that from the five probes (Figure 1) only probe #5 was bound by heterodimers of TWIST1, TWIST2, and Q119X with E12 (Figure 7). The binding of these heterodimers was confirmed by specific competitions using the CH22700EA double-stranded oligo and the SP1 oligo as a non-specific competitor. This finding indicates that the TWIST2 Q119X mutant protein can bind the probe #5 DNA, but only as a heterodimer with E12. The supershift reaction for the TWIST2 Q119X/E12 heterodimer inhibited the DNA–protein complex formation, as had been seen before in EMSAs using an oligonucleotide derived from an E-box located in a conserved region of the human TNFα gene promoter [7]. Even though the TWIST2 Q119X mutant protein can bind as a heterodimer, it may fail to recruit the cofactors necessary for repression of *CHRDL1* gene expression [28].

**Figure 8:**
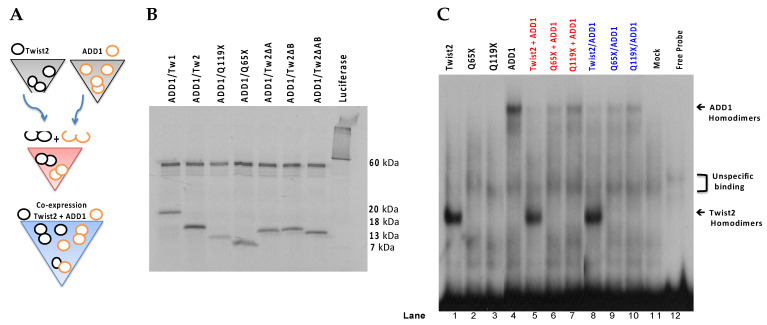
The N-terminus of TWIST2 is involved in SREBP1c’s reduced DNA-binding activity. (**A**) Diagram of how proteins were synthesized in vitro through coupled transcription/translation for the binding reactions to be used in electrophoretic mobility shift assays (EMSA). (**B**) Western Blot of SDS-PAGE of TnT reactions of co-expressed TWIST proteins and SREBP1c, detected with an anti-Myc antibody. Luciferase was used as a positive control for protein synthesis and as a negative control (mock) for EMSA binding reactions. (**C**) EMSA analysis for determining the DNA binding ability of each protein to the DNA. Mock reaction represents the firefly luciferase negative control. Free probe represents unbound labeled DNA.

To determine whether TWIST2 uses its N-terminus to interact with SREBP1c and reduce its ability to bind to DNA, we conducted EMSA assays with C-terminus-Myc/His-tagged SREBP1c and/or N-terminus-Myc-tagged TWIST2 or N-terminal-Myc-tagged TWIST2 mutant proteins (Figure 8). To favor homodimer formation (Figure 8A), each protein was individually produced by in vitro transcription and translation (TnT) (black colored test tubes). The red color test tube represents test tubes used for the binding reaction between homodimers used for EMSA in which 1 µL of each protein that was individually synthesized was mixed for a total of 2 µL (lanes 5–7 in Figure 8C). Since each protein is presumably present as homodimers, it decreases the chances of interacting with the other protein to form a heterodimer; however, we cannot rule out heterodimer formation when these reactions are mixed. However, homodimers can also be present in these mixed reactions.

EMSA results demonstrate that when each protein is individually expressed to increase the likelihood of homodimer formation (Figure 8B), TWIST2 and SREBP1c are each able to bind to the DNA as homodimers (Figure 8C, lanes 1 and 4). As expected, mutant Q65X is unable to bind to the DNA, as it lacks the basic region needed to contact the DNA, (Figure 8C, lane 2), and the Q119X mutant did not bind to the DNA as a homodimer (Figure 8C lane 3). When each protein is individually expressed and then mixed together in the same binding reaction with SREBP1c, this reduces the likelihood of both proteins interacting with each other since we expect that homodimers predominate in these TnT reactions (Figure 8A, red color tube and Figure 8C, lanes 5–7). It can be observed that when TWIST2 homodimers are bound to the DNA, the DNA-binding ability of SREBP1c is undetectable (Figure 8C, lane 5); but when SREBP1c is mixed with TWIST2-Q65X or Q119X, SREBP1c can bind the DNA as a homodimer, although in much reduced amounts (Figure 8C, lanes 6 and 7). This suggests that WT TWIST2 and SREBP1c compete for the same binding site. The reduced amounts of SREBP1c homodimers bound to the DNA when Q65X and Q119X are co-expressed with SREBP1c in the same synthesis reaction to favor interaction between both proteins (Figure 8C, lanes 6–7 versus 9–10) suggests that the N-terminus of TWIST2 interacts with SREBP1c. Notably, the addition or co-expression of TWIST2 Q65X mutant protein caused the highest reduction of SREBP1c homodimer binding detected when compared to the TWIST2 Q119X mutant.

Based on these results and, in agreement with previous studies [26], we can rule out that TWIST2 uses its basic and HLH regions to interact with SREBP1c, as it appears that the region containing only the N-terminus is mainly responsible for this interaction, supporting our hypothesis.

### 3.6. TWIST2 Uses Its Second Conserved Sub-Motif (SEEE) to Regulate the DNA-Binding Activity of SREBP1c

**Figure 9:**
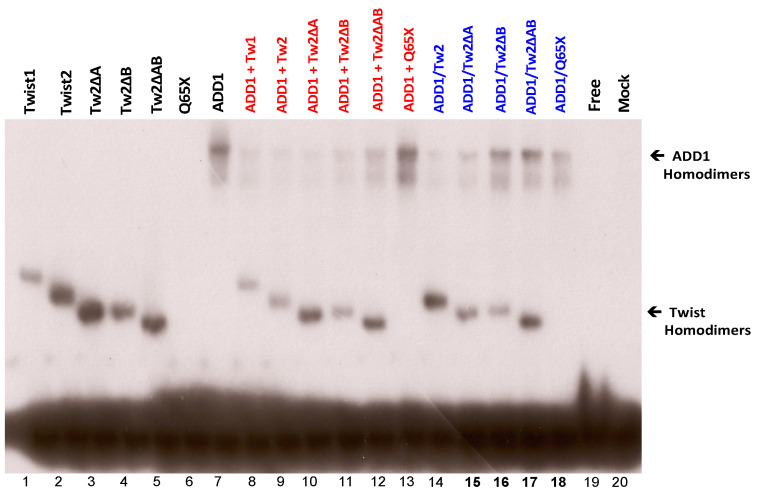
TWIST2 uses its second conserved sub-motif (SEEE) to regulate the DNA-binding ability of SREBP1c in vitro. EMSA analysis was carried out to determine the DNA-binding ability of each protein, as well as to detect how co-expression of TWIST2 or its mutant forms, alter the DNA binding activity of the SREBP1c transcription factor to the *CHRDL1* gene probe #5 region.

In previous work, we described a highly conserved sequence motif that had not been previously described found in the majority of Twist1 and Twist2 vertebrate proteins, particularly amongst the mammalian class where they appear strictly conserved [43]. The motif sequence for Twist1 in mammals is SSSPVSPADDSLSNSEEE, while for mammalian Twist2 sequences, the motif is SSSPVSPVDSLGTSEEE. Since TWIST2 appears to use its N-terminus to regulate the DNA-binding activity of SREBP1c, we wanted to determine if this was mediated via one of the two conserved sub-motifs, which were predicted to be associated with protein binding. Therefore, we hypothesized that TWIST2 uses the conserved sub-motifs sequences to mediate the DNA binding of SREBP1c and that the removal of them would allow SREBP1c to bind to the DNA, possibly due to reduced interaction with TWIST2. We generated mutant TWIST2 expression constructs where we removed the first sub-motif SSSPVSP (TWIST2ΔA), the second sub-motif SEEE (TWIST2ΔB), or both sub-motifs (TWIST2ΔAB) (Supplementary Figure S1A). EMSA results demonstrated that when each of the N-terminal TWIST2 deletion mutant proteins are individually expressed to favor homodimer formation, they still retain the ability to bind to the DNA as homodimers (Figure 9, lanes 3, 4, and 5). When each N-terminus deletion TWIST2 mutant protein is individually expressed, and then mixed with individually expressed SREBP1c in the same binding reaction (Figure 9, red colored reactions), a reduction in the amount of SREBP1c homodimers that bind to the DNA is observed when mutant N-terminal TWIST2 proteins are bound to the DNA (Figure 9, lanes 10, 11, and 12). As with TWIST2, this suggests that N-terminus mutant TWIST2 proteins also compete with SREBP1c for DNA binding to this DNA probe. However, when Q65X is individually expressed and mixed with SREBP1c homodimers in the same binding reaction, SREBP1c is able to bind to the DNA since there are no TWIST molecules bound to the DNA (Figure 9, lane 13). When each of the N-terminus mutant proteins are co-expressed with SREBP1c in the same synthesis reaction to favor interactions between both proteins and favor the formation of heterodimers (Figure 9), it can be observed that upon removal of the conserved sub-motifs, SREBP1c is able to bind to the DNA as a homodimer, although the detected complexes have weaker signals than when only the SREBP1c TnT protein reaction is added (Figure 9, lanes 15, 16, and 17). On the other hand, when co-expressed with Q65X, the amount of SREBP1c homodimers bound to DNA is reduced (Figure 9, lane 18), suggesting formation of an SREBP1c/Q65X complex that lacks DNA binding ability. This provides further evidence that the N-terminus alone, and not the basic or HLH regions of TWIST2, is enough for regulating the DNA binding activity of SREBP1c. These results suggest that the region used by TWIST2 to mediate SREBP1c’s DNA binding is the amino-terminus, particularly through the second sub-motif (SEEE), in agreement with our hypothesis.

### 3.7. The Glycine-Rich Regions Present in TWIST1 Influence Its Interaction with SREBP1c

**Figure 10:**
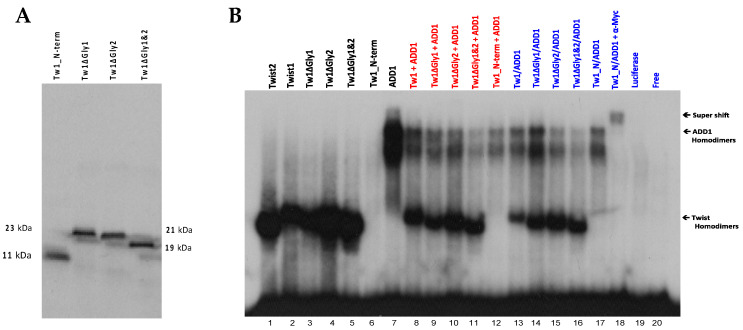
Production of TWIST1 and deletion mutant TWIST1 proteins using TnT reactions and assessment of DNA binding of TWIST1 proteins and SREBP1c to the *CHRDL1* gene probe #5 region. (**A**) To confirm protein expression and stability, duplicate in vitro TnT reactions were analyzed by SDS-PAGE and autoradiography of proteins labeled with ^35^S-Methionine. (**B**) EMSA analysis of TWIST1 and deletion mutants. Homodimers mixed reactions (reactions colored in red): Co-expressed reactions (reactions colored in blue). Addition of anti-Myc antibody to confirm the presence of the SREBP1c-DNA complexes. Firefly luciferase (lane 19) was used as a negative control for EMSA. Free probe (lane 20) represents labeled DNA not bound by protein.

Since the TWIST2 protein but not TWIST1 was described as an interacting partner of *SREBP1c*, we hypothesized that the glycine-rich regions might be used by TWIST1 to interact with proteins that are not bound by TWIST2 and that the glycine-rich motifs present in the TWIST1 N-terminal region might not allow proper interaction with SREBP1c, which would then allow SREBP1c DNA binding to regions like the *CHRDL1* probe #5. To determine whether the sequence difference in the N-terminal region of TWIST1 accounts for the functional difference in protein–DNA interactions with SREBP1c, we generated TWIST1 mutants lacking the first glycine-rich region (Twist1ΔGly1), the second glycine-rich region (Twist1ΔGly2), or both glycine-rich regions (Twist1ΔGly1&2) (Supplementary Figure S1B). In addition, to compare the N-terminal region of TWIST1 with that of TWIST2, we created a TWIST1 mutant protein that contains only the N-terminal region (T108X) and lacks the basic, HLH, and C-terminal domains. Prior to conducting EMSA assays, the proteins were synthesized through in vitro transcription and translation, and the expression and stability of the proteins were confirmed by either SDS-PAGE or Western Blot analysis, resulting in bands of expected weight and intensity (Figure 10A).

The results of EMSAs with TWIST1 and TWIST1 deletion mutants (alone or with SREBP1c) and labeled probe #5 are shown in Figure 10B, where we demonstrate that when TWIST1, SREBP1c and each of the glycine region deletion mutant proteins are individually expressed to increase the formation of homodimers, they all bind the DNA as homodimers with the exception of the Twist1_N-terminus (T108X), which, like the TWIST2-Q65X mutant that lacks the bHLH and C-terminal regions, does not bind DNA. (Figure 10B, black colored reactions, lanes 2–7). When TWIST1 or glycine region deletion-mutant proteins are individually expressed as homodimers, and then mixed with individually expressed SREBP1c homodimers in the same binding reaction (Figure 10B, red colored reactions), binding of TWIST1 to the DNA does not prevent SREBP1c binding (lane 8), which suggests that they do not compete for DNA binding. When we mix Twist1ΔGly1 or Twist1ΔGly2 homodimers with SREBP1c homodimers, even though SREBP1c still binds to the DNA, the amount of SREBP1c-DNA complexes is reduced (lanes 9 and 10 versus lane 8), which suggests that SREBP1c is now competing with TWIST1 glycine-rich mutants for DNA binding. Removal of both glycine-rich regions (making this TWIST1 mutant more similar to TWIST2) drastically reduces the amount of SREBP1c homodimers bound to the DNA (Figure 10, lane 11 versus lane 8). This suggests that SREBP1c binding to the DNA is now being out-competed by mutant TWIST1ΔGly1&2, in agreement with our previous results for TWIST2. Upon mixing SREBP1c with a TWIST1_N-terminus T108X mutant, the amount of SREBP1c homodimers bound to the DNA increase (lane 12). In the co-expression reactions (Figure 10B, blue colored reactions), it can be observed that SREBP1c still binds to the DNA in the presence of TWIST1 (lane 13); however, the reduced amount of TWIST1 and SREBP1c homodimers bound to the DNA suggests that there may be a weak interaction between TWIST1 and SREBP1c. Interestingly, when we co-express TWIST1ΔGly1 with SREBP1c, removal of the first glycine-rich region increases the DNA binding of SREBP1c even further(Figure 10, lane 14). Co-expression of SREBP1c with Twist1ΔGly2 reduces the binding of SREBP1c to the DNA (Figure 10, lane 15), while co-expression with Twist1ΔGly1&2 reduces it even further (lane 16). When we co-express SREBP1c with only the N-terminus region of Twist1, the amount of SREBP1c homodimers bound to the DNA increases again (Figure 10, lane 17), which suggests that the N-terminus of TWIST1 may interact with a fraction of SREBP1c and prevent DNA binding, since the intensity of the SREBP1c homodimer band in that reaction is less than when SREBP1c is expressed alone. Based on these results, it can be inferred that the presence of the glycine-rich regions in the N-terminus of TWIST1 influences the interaction of TWIST1 with SREBP1c, as hypothesized.

### 3.8. SREBP1c and the TWIST2 Q119X Mutant Protein Activate the Luciferase Reporter but Wild Type TWIST2 Blocks SREBP1c-Mediated Activation

**Figure 11:**
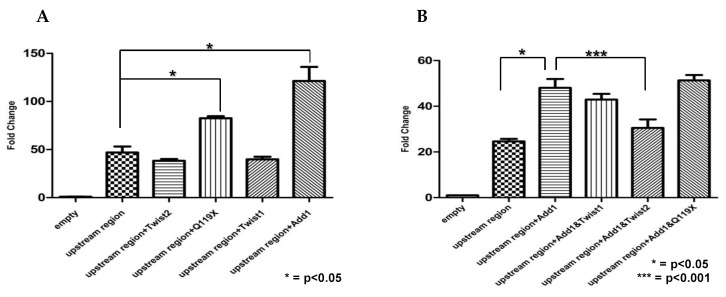
Luciferase reporter gene assay of the -3KCHRDL1-pGL4 construct to assess the effect of different bHLH proteins on the *CHRDL1* gene upstream region. Transient transfection of GM00637 SV-40 transformed human fibroblast cells was performed as described in the Section 2. (**A**) The upstream region of CHRDL1 (from −2751 to +85) was able to drive expression of the -3KCHRDL1-pGL4 reporter construct, indicative of promoter activity. TWIST1 and TWIST2 reduced the basal expression of the reporter gene, but this reduction was not significant. Both SREBP1c and TWIST2 Q119X were able to significantly increase luciferase activity, indicating that both had an activating effect. (**B**) Luciferase reporter gene assays to assess the effect of TWIST1, TWIST2 and Q119X on SREBP1c-mediated activation. TWIST2 but not TWIST1 was able to significantly block activation by SREBP1c, suggesting that this inhibition may be specific for TWIST2. The TWIST2 Q119X mutant was unable to block SREBP1c activation, as expected. Statistical analysis for experiments presented in A and B were performed using Student’s *T*-test (N = 3). Error bars represent the standard error of the mean (SEM).

To determine the effect of these proteins in the transcriptional regulation of *CHRDL1* we performed reporter gene assays using a luciferase construct containing the upstream region of *CHRDL1* (from −2752 to +85). When transfected into human skin fibroblasts, the reporter construct containing the upstream region showed luciferase activity indicating that this region was able to drive the expression of the luciferase gene (Figure 11). The expression of luciferase was measured in the presence of overexpressed transcription factors. Neither TWIST1 nor TWIST2 were able to significantly repress luciferase gene expression. Luciferase expression significantly increased in the presence of SREBP1c, as expected, as well as when the TWIST2-Q119X mutant protein was overexpressed (Figure 11A). We then assessed the effect of TWIST1, TWIST2 and the TWIST2-Q119X mutant when co-expressed with SREBP1c. As seen in Figure 11B, SREBP1c activation of the reporter gene was significantly blocked by TWIST2, but not by TWIST1 or TWIST2-Q119X, which may explain why *CHRDL1* gene expression is elevated in patient dermal fibroblasts [29]. Whether that requires binding by TWIST2 homodimers to the upstream *CHRDL1* gene region we tested by EMSAs (probe #5), which the homodimers of the Q119X mutant protein failed to do, or formation of heterotrimeric complexes that may or may not bind DNA, deserves further study.

### 3.9. In HeLa Cells, TWIST2 and Its N-Terminal Mutant Forms Reduce the Transactivating Activity of SREBP1c

As already mentioned in the introduction, the *CHRDL1* gene is a differentially up-regulated gene in Setleis syndrome patient dermal fibroblast cells of [29]. Hence, we hypothesized that TWIST2 acts as a repressor in *CHRDL1* gene expression, since the truncated TWIST2-Q119X mutant protein expressed in patients appears to lack the capacity to repress its expression. In order to assess the consequences of the different proteins binding to the human *CHRDL1* gene upstream region we performed reporter gene assays. Luciferase reporter gene assays using HeLa cells demonstrated activation of the luciferase reporter gene by the *CHRDL1* gene upstream region, confirming promoter activity (Supplementary Figure S4A, promoter). When each protein is expressed alone, neither WT TWIST2 nor mutant forms TWIST2ΔA and TWIST2ΔB caused any significant difference in the basal expression of the reporter gene, with the exception of mutant TWISTΔ2AB, which significantly lowered it (Supplementary Figure S4A, white bars relative to promoter). This observation was expected, as it is consistent with reports of TWIST2 as a transcriptional repressor [26]. On the other hand, ectopic expression of SREBP1c alone increased the expression of the reporter gene. This observation was also expected due to reports that demonstrate SREBP1c′s role as a weak activator [26,48]. Co-expression of SREBP1c and mutant form TWIST2ΔA decreased the expression of the reporter gene, while co-expression of SREBP1c with WT TWIST2, TWIST2ΔB or TWIST2ΔAB significantly decreased it when compared to the promoter (Supplementary Figure S4A, black bars relative to promoter). To assess the effect of TWIST2 and N-terminal mutant forms on SREBP1c-dependent activation, we compared the luciferase activity of the co-transfected reactions relative to SREBP1c′s transactivating activity. As can be observed in Supplementary Figure S4B, co-expression of TWIST2, TWIST2ΔB, or TWIST2ΔAB significantly suppressed SREBP1c-dependent activation of the *CHRDL1* gene promoter reporter gene. These results imply a repressor role for TWIST2 and its mutant *N*-terminal forms.

**Figure 12:**
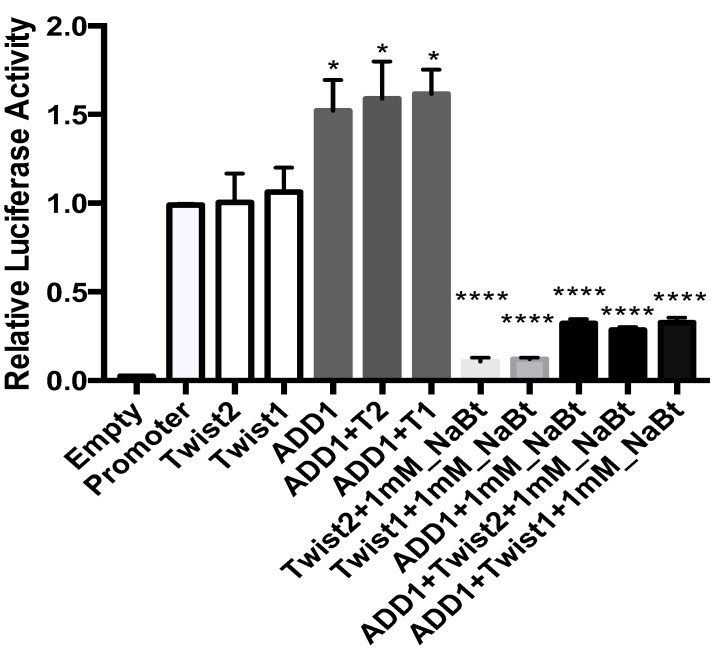
The expression of the -3KCHRDL1-pGL4 reporter gene construct is significantly reduced with sodium butyrate (NaBt) treatment. Approximately 1 × 10^6^ COS7 cells were transiently transfected or co-transfected with the indicated expression vectors. Transfection media were removed after 4 h of incubation and replaced with media. Sodium butyrate (1 mM) was added directly to cells 24 h prior to cell lysis and incubated overnight. Luciferase activity was normalized relative to the basal promoter activity. Co-expression of the transcription factors increased the expression of the reporter gene. Addition of NaBt significantly reduced the expression of the reporter gene. Transfection experiments were performed in duplicate and repeated independently five times (N = 5). Statistical analysis was performed using one-way ANOVA followed by unpaired Student’s *T*-test. Error bars represent standard error of the mean (SEM). * = *p* < 0.05; **** = *p* < 0.0001.

Some mechanisms of repression exerted by TWIST1 and TWIST2 involve the recruitment of histone deacetylases (HDACs) [27]. To determine the effect of histone deacetylation on the observed expression signal, COS-7 cells were transfected and treated with sodium butyrate (and HDAC inhibitor) 24 h prior to cell lysis to prepare extracts for luciferase activity assays. As shown in Figure 12, when each construct is expressed alone, TWIST1 and TWIST2 exert no significant change in the expression levels of the reporter gene when compared to basal expression, but SREBP1c significantly increased it. Co-expression of TWIST1 or TWIST2 with SREBP1c increased the basal levels of the reporter gene relative to the promoter, but it appears that the activation of the reporter gene is mainly mediated by SREBP1c. To eliminate the possibility that the mechanism of repression involves HDACs as previously reported [26], we added sodium butyrate (an HDAC inhibitor). We were expecting to see increased levels of reporter gene expression upon addition of sodium butyrate, but instead we saw a very significant decrease in the expression of the reporter gene, more so in the transfections in which the proteins were expressed alone than when co-expressed together with SREBP1c. These results suggest that recruitment of HDACs is not involved in the transcriptional repression observed with TWIST proteins.

## 4. Discussion

We selected the *CHRDL1* gene as a TWIST2 target gene based on it being the most up-regulated in Setleis syndrome patients who express the Q119X truncated mutant protein. This gene appeared to be a good candidate gene for studying TWIST2 function in repressing gene expression through protein–protein interactions and, possibly, the recruitment of HDACs. Though evidence of this type of protein–protein interaction still comes from studies like the ones performed on the inactivation of the p53 protein [49,50], our data have directed us to a more recent role of Twist2 as a bi-functional protein able to repress or activate genes depending on partner choice for dimerization. This role was established by Spicer’s group [51] for Twist1 and expanded to Twist2 by the work of Sharabi et al., Laursen et al. [52,53] and Franco et al. [28]. The TGFβ signaling pathway has an important role in patterning during craniofacial development, and thus changes in the expression of BMP antagonists like CHRDL1 could have an impact in the developmental program [54].

Our working hypothesis was that TWIST2 repressed the *CHRDL1* gene and that the mutant form of TWIST2 harbored by Setleis syndrome patients failed to repress it. We first analyzed the upstream region of the human *CHRDL1* gene 5′ of the transcription start site by TESS. The finding that it contained several E-boxes was expected, but it was interesting to observe putative binding sites for proteins known to interact with TWIST2 (Figure 1). A similar pattern of binding sites was reported for the pro-inflammatory cytokines IL-1β and TNF-α [16]. We further analyzed for conservation of the upstream region of the *CHRDL1* genes using mVISTA. This analysis revealed that the *Chrdl1* gene upstream regions are primarily conserved in mammals, including most of the binding sites we focused on, which were found by TESS analysis (Supplementary Figure S2).

When in vitro synthesized proteins were assessed for DNA binding to these conserved sites, we saw that the main binding sites in the upstream region of *CHRDL1* are contained in probes #1 and #5. The other putative sites should be considered in future experiments for other bHLH proteins based on their sequence conservation, such as Hand1 and Hand2, as they are able to bind both E-boxes and D-boxes [55]. When probe #1 was assessed for binding by EMSA, we found that only E12 was able to cause a mobility shift. Since this site is close to the transcription start site, E12 homodimers may be important for interaction with the transcription machinery, since they have been reported to act mainly as activators [56].

EMSA probe #5 gave more interesting results, where we observed binding by TWIST1, TWIST2, and SREBP1c homodimers. For this probe, we consistently observed that TWIST2 appeared to bind more strongly than TWIST1. It is of note that, since similar quantities of TWIST1 and TWIST2 were synthesized (Supplementary Figure S3 and Figure 8), the higher intensity observed for the TWIST2 shift compared to TWIST1 suggests that TWIST2 has a higher affinity to the E-boxes in probe #5. Nonetheless, these EMSA experiments do not allow for the calculation of binding affinities, since they were carried out using high probe quantities that cause saturation. Another caveat for affinity estimation is that the individual proteins are very difficult to quantify within the reticulocyte extracts, though the SDS-PAGE fluorography and Western blot analyses provide information on the relative levels of each protein.

By designing small oligonucleotides based on probe #5 (Figure 4, Figure 5 and Figure 6), we were able to define, through EMSAs, the binding region for Twist2 and SREBP1c to sites −2661 and −2648. SREBP1c has been reported to be important in the regulation of lipid metabolism and adipocyte differentiation (26). The finding that it can bind the *CHRDL1* gene upstream sites −2661 and −2648 indicates that SREBP1c may regulate *CHRDL1* gene expression (Figure 5). In addition, EMSA data suggest that TWIST2 competes with SREBP1c for binding the −2648 site (Figure 6). It is interesting that TWIST2 apparently prefers to bind the −2648 site even when the −2661 site has the same E-box consensus sequence (Figure 5). This could be due to flanking sequence differences between the two sites, which should be further examined in future experiments. The finding that TWIST proteins were unable to bind to the −2611 site should not prevent future experiments with other E-box binding proteins. This site might have an important regulatory role due to its high degree of conservation in mammals (Supplementary Figure S2C). Taken together, we propose that TWIST2 binds to this region and blocks binding of SREBP1c, possibly by a physical interaction, as described by Lee et al. [26].

Even though SREBP1c can bind to both E-boxes, it seems less likely that if TWIST2 (or one of its mutant N-terminal forms) bind as homodimers to one E-box, SREBP1c homodimers bind to the other E-box, as the formation of a third band representing a SREBP1c-TWIST-DNA triple complex was not observed in some of our EMSAs (Figure 9). We do not rule out the possibility that this could be happening in vivo. Instead, it appears that TWIST2 and its mutant N-terminal forms compete with SREBP1c for the second E-box. Nonetheless, Chang et al. [57] showed that the double E-box Twist-binding motif is highly conserved from *Drosophila* to humans and specifically recognized by the Twist family of bHLH transcription factors. In addition, they showed through molecular modeling that in a double E-box, it is possible for two bHLH dimers to bind both E-boxes, particularly if there is a spacing between the two E-boxes of exactly five-nucleotides as this would allow a full turn of the DNA double helix between the two E-boxes, allowing the conserved base pairs of each E-box to face the same orientation in the DNA [57]. This spatial direction would allow the alignment of two dimers facing the same orientation on the DNA, hence increasing protein–protein interactions between them via hydrophobic interactions and hydrogen bonding, and the formation of a stable ternary complex [57]. If the spacing between both E-boxes is less or more than five nucleotides, this would cause steric clashing of the loops or decreased contact between loops, respectively [57]. However, the authors suggest that the five nucleotides spacing between the two E-boxes is optimal for the stable binding of two Twist1/E47 heterodimers.

Based on our EMSA results though, it seems more likely that if TWIST homodimers are bound to one E-box, TWIST homodimers bind to the other E-box; and if SREBP1c homodimers bind to one E-box, then SREBP1c homodimers bind the other E-box. Therefore, we suggest that perhaps a double E-box separated by six-nucleotides allows more stable binding of two TWIST/TWIST homodimers. We do not rule out the possibility that perhaps in vivo, it may also allow some binding of TWIST/E-protein heterodimers; or the binding of different types of homodimers. For example, if TWIST homodimers bind to the second E-box, then SREBP1c homodimers could bind to the first E-box, but we did not detect this type of complex in our in vitro binding assays.

Although TWIST1 has been more extensively studied than TWIST2, not enough studies have compared whether the sequence differences found in the N-terminus of TWIST1 and TWIST2 account for their functional difference in interactions with a particular protein. SREBP1c was shown to interact with TWIST2 [26]; however, TWIST1 was not reported as an interactor of SREBP1c. Therefore, we decided to determine whether TWIST1 was able to interact with SREBP1c and reduce its DNA binding ability, as observed with TWIST2; or if it was not able to due to the sequence differences found in their amino terminal regions.

In contrast to what was observed with TWIST2, the N-terminus of TWIST1 interacts less with SREBP1c (Figure 9 vs. Figure 10). This could be due to the presence of TWIST1′s glycine-rich regions. In addition, it appears that TWIST1 does not compete for DNA binding with SREBP1c since it was observed that in the presence of TWIST1, both proteins were able to bind to the DNA as homodimers (Figure 10). The reduced amounts of TWIST1 and SREBP1c bound to the DNA upon co-expression suggests some type of interaction between both proteins and removal of the first glycine-rich region reduces it, allowing more of each protein to bind the DNA. Upon removal of the second glycine region though, SREBP1c DNA binding is reduced, and it decreased even more when both regions were removed. This indicates that there is interaction between both proteins, and although we could infer that mutant TWIST1ΔGly1&2 could be using the SEEE sub-motif to interact with SREBP1c, as does TWIST2, this would have to be confirmed by generating additional TWIST1 deletion mutants. However, because there was barely any difference in the amounts of TWIST1 homodimers bound to the DNA (Figure 10 lanes 2 and 7 versus lane 13), it is most likely that the mechanism by which the mutants TWIST1ΔGly2 and Twist1ΔGly1&2 reduce SREBP1c binding to the DNA is by out-competing SREBP1c for DNA binding, as observed with TWIST2.

Upon performing luciferase reporter gene assays, we saw activation of the luciferase reporter by the *CHDRL1* gene upstream region, confirming that this region promotes transcription. The luciferase assays also confirmed that SREBP1c was able to activate luciferase gene expression driven by the *CHRDL1* gene upstream region (Figure 11 and Supplementary Figure S4). These findings suggest that *CHRDL1* is a novel target gene of SREBP1c and should be considered when studying the role of SREBP1c in BMP signaling. Interestingly, overexpression of the TWIST2-Q119X mutant form increased the reporter gene expression. This was surprising given that the TWIST2 Q119X mutant protein did not bind to the upstream region of the *CHRDL1* gene. We do not think that the binding observed for TWIST2-Q119X when overexposing the EMSA gel in Figure 2 is relevant since it is nonspecific. Neither TWIST1 nor TWIST2 cause any difference in reporter gene expression, which was unexpected for TWIST2, where we thought that some significant decrease in luciferase activity would occur when TWIST2 was added (Figure 11A). Nonetheless, TWIST2 was able to repress the activation caused by SREBP1c (Figure 11B) as previously reported [26]. As expected, the Q119X Twist2 mutant protein was unable to block SREBP1c-mediated activation (Figure 11B). Though it could be argued that the failure of the Q119X mutant protein to block SREBP1c is due to the lack of the C-terminus, it is important to point out that TWIST1 was not able to block SREBP1c, which suggests that this inhibition may require the N-terminus, where TWIST1 and TWIST2 differ the most [43]. This mechanism of inhibition should be further explored to determine if it is really a TWIST2-exclusive event. The fact that TWIST2 interacts with the N-terminus of SREBP1c, not with its bHLH domain, is noteworthy [26]. Taken together, these findings lead us to speculate about the possible exclusive interaction between SREBP1c and TWIST2, which could be dependent of its N-terminus since TWIST1 and TWIST2 have nearly identical bHLH and C-terminal regions. Further experiments are needed to assess this possibility, which could lead to characterizing the inhibition of SREBP1c as a non-redundant function exclusive to TWIST2.

Up until this point all the EMSAs discussed only considered homodimers of the different proteins, since we tried to set up binding reactions with more than one protein at a time but only observed homodimeric binding. We performed the in vitro coupled transcription/translation reactions (TnT) by simultaneously adding the two expression constructs to generate the proteins for which we wanted to see heterodimers. We reassessed all five EMSA probes to see if there was binding of heterodimers. Interestingly, we only detected binding to probe #5 (Figure 7), where we observed binding of Twist1/E12 heterodimers, Twist2/E12 heterodimers, and, to our surprise, Q119X/E12 heterodimers. Our group had previously shown that the TWIST2-Q119X mutant protein was able to form homodimers and heterodimers with E12 [28]. This finding agrees with EMSA results previously published by Tukel et al. [7], where the TWIST2-Q119X mutant form was shown to bind the TNF-α E-box as a heterodimer with E12. This result, together with the finding that the TWIST2-Q119X activated the luciferase reporter, suggests that the mechanism of regulation employed by TWIST2 on the *CHRDL1* gene is similar to the one reported for Twist1 in the regulation of thrombospondin [51,58]. The work performed by the Spicer lab proposes a mechanism of regulation that is dependent upon dimer choice, where Twist1 homodimers might activate or repress a target gene while the Twist1/E12 heterodimers cause the opposite result. Based on these findings we could speculate about the possible scenarios in normal versus SS patient cells. Since the bHLH dimer pool inside cells is important to dictate the transcriptional outcomes, we hypothesize that, in normal cells, an increase in the homodimer to heterodimer ratio of wild-type TWIST2 would repress *CHRDL1* gene expression. The opposite would also be true; the decrease in the homodimer to heterodimer ratio when TWIST2 is mutated would lead to activation of the *CHRDL1* gene. In the case of Setleis patient cells, *CHRDL1 gene* expression would be active independent of the homodimer to heterodimer ratio since only the TWIST2-Q119X/E12 heterodimer would be functional (Figure 7). Though this is a tempting explanation, we cannot rule out the possibility of TWIST2-Q119X forming heterodimers that lack DNA binding capacity, in a manner similar to the Id proteins. To better understand the mechanism of regulation, it would be necessary to identify binding partners for TWIST proteins and to carry out EMSAs, luciferase and chromatin immunoprecipitation (ChIP) assays using tethered dimers of TWIST proteins with their identified partners. Another possibility is that the TWIST2-Q119X mutant has de novo functions that have yet to be described, which would make the TWIST2-Q119X mutant a gain-of-function mutation.

**Figure 13:**
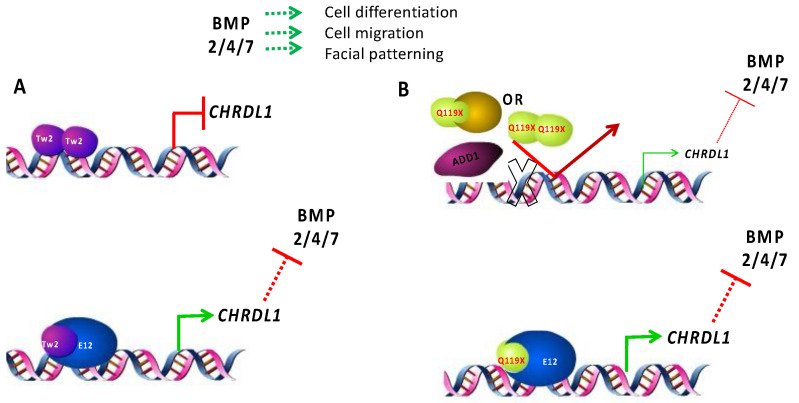
Adaptation of the model reported by the Spicer group of the bi-functional role of TWIST2 in the regulation of the *CHRDL1* gene. (**A**) As seen for thrombospondin [51] homodimers of TWIST2 might repress *CHRDL1* gene expression while TWIST2/E12 heterodimers might activate expression. The expression state of the CHRDL1 gene at a given moment would be determined by the abundance of either homo- or heterodimers. (**B**) In Setleis syndrome patients harboring the Q119X, the mutant homodimers appear to be non-functional. That is not the case for the Q119X/E12 heterodimers, which are able to activate the *CHRDL1* gene. Hence, activation of *CHRDL1* might occur even in situations where the homodimer to heterodimer ratio is high. Two alternatives to this model are that: (1) Q119X homodimers bind weakly and could be easily displaced by SREBP1c leading to CHRDL1 activation, and (2) Q119X might sequester other HLH repressors in a similar manner to the Id proteins.

The failure to correctly regulate and fine-tune the expression of *CHRDL1* gene expression could explain some of the phenotypic features observed in patients. BMPs and BMP antagonists are secreted proteins that exert their function in a dose-dependent manner [59]. This concentration equilibrium has been shown to be important in the context of facial development [60]. Here we propose a model of the action of TWIST2 on the *CHRDL1* gene, which leads to modulating BMP signaling (Figure 13). In normal individuals, TWIST2 can effectively act as a molecular switch between activation or repression of BMP signaling through the control of *CHRDL1* gene expression. Given that CHRDL1 is a BMP2/4/7 antagonist, we could speculate that during critical times in the formation of facial structures in the Setleis patient embryos, the presence of CHRDL1 would lead to hypo-activation of the BMP signaling pathways and disruption of the BMP spatiotemporal gradient. This idea warrants a careful examination of the available data for the different mouse models concerning facial malformations, and to evaluate expression patterns of the BMPs and BMP antagonists in the tissues that give rise to the face during development. Other members of the TGF-β/BMP super-family should also be considered in order to determine where these molecules fit in our proposed model as other genes involved in BMP signaling were also found to be differentially regulated on Setleis syndrome patients [29].

There have been reports that demonstrate the role of TWIST2’s N-terminus and C-terminus in the trans-repression of other factors as in the case of MyoD, MEF2 [61], SREBP1c [26], and RunX2 [9]. However, most of the studies conducted involve TWIST2′s C-terminus and not enough attention has been given to its N-terminus. SREBP1c was found to be an interactor of TWIST2, and although the authors determined that TWIST2 interacted with SREBP1c through its N-terminus, the region used by TWIST2 was not described [26]. We used the TWIST2-Q65X mutant protein that only contains the N-terminus region to determine whether the N-terminus of TWIST2 is involved in SREBP1c′s reduced DNA binding ability, and observed that upon co-expression with SREBP1c, the amount of SREBP1c bound to the DNA was greatly reduced (Figure 9 Lane 18), implying that the TWIST2 N-terminus alone and not the basic or HLH regions is involved. We conducted deletion mutagenesis of the conserved sub-motifs predicted to have a role in protein binding and found that the second sub-motif SEEE was mainly responsible for regulating the ability of SREBP1c to bind to the DNA.

Also, it can be noted that the amounts of TWIST2ΔA protein bound to DNA when co-expressed with SREBP1c versus the amount bound in the homodimers reaction is reduced. This could be because there is more of TWIST2ΔA interacting with SREBP1c, and this TWIST2ΔA/SREBP1c complex cannot bind DNA, but its formation reduces the amount of TWIST2ΔA homodimers available for binding the probe. On the other hand, the reduced intensity of the TWIST2ΔB band, both in the homodimeric reactions and when co-expressed with SREBP1c, could be because this particular mutant has lower binding affinity towards the DNA than the TWIST2ΔA or Twist2ΔAB mutants.

The probe-SREBP1c and probe-TWIST2ΔAB complexes are specific for both proteins, as confirmed by the competition and super-shifts observed upon the addition of specific antibodies for each protein. Because a DNA–SREBP1c–Twist2ΔAB or DNA–SREBP1c–Q65X triple complex was not observed, and the amounts of SREBP1c bound to DNA are greatly reduced when co-expressed with TWIST2-Q65X, it can be inferred that TWIST2 reduces the amount of SREBP1c bound to the DNA by directly binding to it, and perhaps titrating it away by forming a complex that cannot bind DNA, in a similar way to the mechanism of the inhibitory HLH (Id) proteins [62], which was also suggested previously [26]. Similar reports describe this type of mechanism in the regulation of MyoD and MEF2 by TWIST2 [61,63]. Interestingly, we were expecting to see more binding of SREBP1c to the DNA when co-expressed with mutant TWIST2ΔAB as removal of the sub-motifs would reduce the interaction between SREBP1c, allowing it to bind to the DNA. However, we saw reduced amounts of SREBP1c bound to the DNA. Therefore, it appears that even though removal of the sub-motifs reduces the interaction between SREBP1c and TWIST2ΔAB, this could allow SREBP1c to bind to the DNA, but SREBP1c still has to compete with Twist2ΔAB for DNA binding to the *CHRDL1* gene region, which contains the −2661 and −2648 E-boxes.

When determining the regulatory effect of TWIST2, mutant forms (TWIST2ΔA, TWIST2ΔB, TWIST2ΔAB) and SREBP1c on the *CHRDL1* gene upstream region using different type of cells, we observed that the magnitude of the effects detected, be it activation or repression, were low, hence, we cannot make mechanistic studies with the results obtained. There are several limitations to this study that could have influenced the obtained results. Perhaps the use of a different cell line such as the C3H10T1/2 cells would have been a more appropriate cell model since they are embryonic cells. Or perhaps induction of gene activation first by, for example, Dexamethasone, which was recently described to regulate the expression of *CHRDL1* [64], would allow better detection of repressive action by TWIST2 proteins. Furthermore, perhaps use of other SREBPs such as SREBP1a or SREBP2 could have shed some light on whether the low signal of expression was in fact due to the weak activating activity of SPREBP1c.

To our surprise, the addition of NaBt significantly decreased the expression of the reporter gene. However, butyrate has been reported to suppress the TGF-β-1 dependent signaling pathway [65]. Matsumoto et al. [65], examined the activation of the SMAD signaling pathway using the (SBE)4-Lux reporter containing a SMAD-binding element. Addition of TGF-β1 stimulated the expression of the reporter gene, but in the presence of 1 or 10 mM butyrate, it significantly decreased TGF-β-dependent activation of SMAD signaling by preventing TGF-β-dependent phosphorylation of SMAD.

## 5. Conclusions

In summary, we found six predicted Twist2 binding sites in the 5′ upstream region of the *CHRDL1* gene and that this region is conserved in mammals. For the site closest to the transcription start site (TSS), we only observed binding of E12 homodimers. For the most upstream site, we observed binding of TWIST1, TWIST2, and SREBP1c homodimers as well as TWIST1/E12 heterodimers, TWIST2/E12 heterodimers, and TWIST2-Q119X/E12 heterodimers. The −2611 site does not appear to be either a TWIST or SREBP1c binding site. SREBP1c can bind both the −2661 and the −2648 sites, while TWIST2 prefers to bind to the -2648 site. TWIST2 was able to compete out SREBP1c from binding these sites and, vice versa, SREBP1c was able to compete out Twist2 binding. In the luciferase reporter gene assays, we saw that overexpression of both SREBP1c and TWIST2-Q119X proteins increased luciferase expression driven by the *CHRDL1* gene upstream region. In addition, wild-type TWIST2 but not the TWIST2-Q119X mutant protein was able to block SREBP1c-mediated activation. Finally, the activation caused by the TWIST2-Q119X TWIST2 mutant protein could be explained by the formation of TWIST2-Q119X/E12 heterodimers, which may be able to bind the most upstream site of the *CHRDL1* gene.

Taken together, these results suggest a mechanism of regulation of the *CHRDL1* gene not considered in our working hypothesis, especially when we assumed that the Q119X mutant form of TWIST2 lacked function based on published reports of TWIST2 as a repressor and previous findings from our group where the Q119X mutant protein was unable to activate the periostin gene [28]. The data included in this study show that the TWIST2-Q119X mutant protein cannot bind to the probe #5 region of the *CHRDL1* gene as a homodimer, but can bind it as a heterodimer. The finding that overexpression of the Q119X mutant caused increased luciferase activity suggests that this mutant protein has some functionality that needs to be further characterized, to determine if this effect is direct or indirect. Future studies could consider the functional differences between TWIST2-Q119X and the other mutant forms harbored by Setleis patients or from patients who have more severe genetic disorders caused by TWIST2 mutations (Barber Say and Ablepharon Macrostomia) [15], which are inherited in an autosomal dominant fashion. The functional comparisons of the different mutants could provide insight into the differences in the pattern of the inheritance and the partial phenotypes seen in heterozygous relatives of Setleis patients [6,12]. We can speculate that the most severe mutant forms of TWIST2 might not be able to compensate with one normal allele, while less severe mutant proteins can in the case of heterozygous individuals. The TWIST2-Q119X mutation could be considered a less severe mutation harbored by Setleis syndrome patients described so far, when compared to the TWIST2-Q65X mutation and the frameshift mutation found in Mexican-Nahua patients [10]. The published results indicate that the TWIST2-Q65X mutant protein cannot form dimers (since it lacks the bHLH and C-terminal regions) and is unable to migrate to the nucleus, while the Q119X mutant can form homodimers and heterodimers with E12 and migrate to the nucleus [28]. In addition, the TWIST2-Q119X mutant protein can bind DNA as a homodimer and as a heterodimer [7,28].

The deficiency of BMP4 signaling or any changes in its spatial and temporal expression in mouse cranial neural crest has been shown to lead to multiple defects in craniofacial muscle and skeletal development, indicative of its crucial role for craniofacial myogenesis and skeletogenesis [54,66]. Craniofacial development is a complex process where cells that form the facial skeleton muscle originate from the cranial neural crest, and migrate from the dorsal neural tube into the facial prominences. Any event that disrupts not only the rate, but also the timing or the extent of the complex cellular signals that aid in their migration, can result in a craniofacial anomaly [66]. The insufficient migration of the neural crest cells into the frontonasal process and first branchial arch observed in Setleis syndrome patients could be due to the antagonistic effect that Chordin-like-1 exerts on BMP4 and could explain the mesodermal dysplasia observed at the bitemporal forceps-like lesions found in the patients. In addition, Table 1 lists the known interactions exerted by TWIST2’s C-terminus region. Since the TWIST2-Q119X mutant protein lacks the C-terminus, these interactions would be affected in the Puerto Rican Setleis syndrome patients. And as can be observed, known interactors are all important proteins that regulate the development of bone and muscle, linking TWIST proteins in cross-talk with other developmental programs during early stages of embryogenesis.

During mouse development, the *CHRDL1* gene is expressed in many tissues, including dorsal root ganglia, gut, condensing cartilages of the skeleton and developing hair follicles, which resembles the mouse expression pattern of TWIST2. Therefore, *CHRDL1* might be involved in the development of appendages such as hair follicles, bones of the face, and even neonate skin. Defects in the *CHRDL1* gene give rise to Megalocornea disease, which is characterized by enlarged anterior eye segments. These patients suffer from corneal degeneration, presenile cataract and glaucoma. Setleis syndrome patients have a thin cornea, which could be due to overexpression of *CHRDL1*. It is interesting how when the *CHRDL1* gene is defective the cornea becomes bigger, and when TWIST2 is defective, the cornea is thin. Further studies on the regulation of the *CHRDL1* gene by TWIST proteins should be carried out in mouse models. Since E12/E47 bHLH proteins are ubiquitously expressed, TWIST2-Q119X could be binding to E12 during the development of facial structures, reducing the amount of E12 that can bind to other bHLH proteins, including TWIST1, causing a change in the dimer pool and favoring the formation of TWIST1/TWIST1 homodimers that may bind DNA, which may cause activation of the *CHRDL1* gene. This mechanism of action would greatly resemble the role of TWIST1 during osteogenesis in which TWIST1, as a heterodimer with E12, represses the FGFR2 gene, and when Id protein levels rise, it forms a complex with E12, sequestering it and leading to TWIST1 homodimerization. As a homodimer, TWIST1 activates FGFR2, which mediates downstream activation of RunX2 [67]. The TWIST2-Q119X mutant could be acting as an Id protein in our model involving the regulation of SREBP1c DNA-binding activity.

Nonetheless, abnormal activation of the Chordin-like-1 gene produces an antagonist of BMP4, which then directly binds to BMP4 and prevents it from binding to its receptor. This causes the SMAD signaling pathway to be hindered and reduced/increase the expression of its target genes involved in development of the facial bone and muscle, and possibly other structures, which deserves further studies using, for example, the mouse TWIST2 knockout model [16].

## Figures and Tables

**Table 1:** Interactions exerted by TWIST2′s C-terminal region that would be affected in Setleis syndrome patients harboring the Q119X mutation.

Twist Protein	Interactor	Domain Required by Twist	Effect	Domain Targeted in Interactor	Cell Line Used	Source
Twist2	Runx2	Twist Box (Last 20 residues)	Inhibition of Runx2 DNA binding	Runt domain	Osteoblasts	[9]
Twist2	MEF2	C-terminus 121–160 aa	Inhibition of MEF2 transactiva-tion activity	Transactivation domain	C3H10T1/2 cells	[61]
Twist2	MyoD	C-terminus	Repression of MyoD transactiva-tion activity	Basic and HLH	C3H10T1/2 cells	[61]

## Data Availability

The data presented in this study are available in the article and the supplementary information.

## Supplementary Materials

The following supporting information can be downloaded at: https://www.mdpi.com/article/10.3390/genes14091733/s1. Figure S1: Diagram of the TWIST2 and TWIST1 N-terminal mutant proteins produced by deletion mutagenesis. Figure S2: Bioinformatic analysis of the 5′ upstream region of the human CHRDL1 gene to identify putative TF binding sites and sequence conservation. Figure S3: SDS-PAGE of in vitro transcribed/translated proteins and Electrophoretic Mobility Shift Assay for probe #1 of the *CHRDL1* gene. Figure S4: Suppression of the transcriptional activity of ADD1/SREBP1c by TWIST2 and its deletion mutant N-terminal forms in HeLa cells. Table S1: Sequences of Primers used in this study for deletion of conserved regions in the TWIST2 coding sequence, amplification of the human CHRDL1 gene upstream region and of the TCF3 gene coding region for production of an expression construct. The primers used to amplify the human *CHRDL1* gene sequences for reporter gene assays contained additional nucleotides to add KpnI and XhoI sites at the ends of the PCR product for directional cloning into the pGL4.14 vector. Sequences of the oligonucleotides used for competition reactions in electrophoretic mobility shift assays are also included, these oligonucleotides were used as double stranded molecules. E-box and SP1 elements (normal or altered) are shown in italics. E-box bases changed are underlined.
