# Supplementary material for: Mechanisms of Regulation of the CHRDL1 Gene by the TWIST2 and ADD1/SREBP1c Transcription Factors
Source: Genes (Basel). 2023 Aug 30;14(9):1733. doi: 10.3390/genes14091733 (PMC10530651; doi:10.3390/genes14091733)
Supplement: Supplementary file 1 [file genes-14-01733-s001.zip › genes-2510428-supplementary.pdf]

Supplementary Table S1. Sequences of Primers used in this study for deletion of conserved regions in the TWIST2 coding sequence, amplification of the human *CHRDL1* gene upstream region and of the *TCF3* gene coding region for production of an expression construct. The primers used to amplify the human *CHRDL1* gene sequences for reporter gene assays contained additional nucleotides to add KpnI and XhoI sites at the ends of the PCR product for directional cloning into the pGL4.14 vector. Sequences of the oligonucleotides used for competition reactions in electrophoretic mobility shift assays are also included, these oligonucleotides were used as double stranded molecules. E-box and SP1 elements (normal or altered) are shown in italics. E-box bases changed are underlined.

Name of Primers/Oligo-Nucleotide	Sequence	Size	Application
Twist2ΔA	FW:<br>5'-CGATGAGGAGGGCGTGGACAGCCTGG-3'<br><br>RV:<br>5'-CCAGGCTGTCCACGCCCTCCTCATCG-3'	26 bases	Deletion Mutagenesis of first sub-motif SSSPVSP
Twist2ΔB	FW:<br>5'-CAGCCTGGGCACCCTCGAGAGGCAGC-3'<br><br>RV<br>5'-GCTGCCTCTCGAGGGTGCCCAGGCTG-3'	26 bases	Deletion Mutagenesis of second sub-motif SEEE
CH3KLuc-FW	5'-ACGGTACCGGAAGGGAAAAGATGGGTGT-3'<br><br>Includes KpnI site (underlined) for cloning	28 bases	PCR forward primer used for Preparation of *-3KCHRDL1-pGL4 Luc* construct
CH3KLuc-RV	5'-ACTCGAGTGCTCACTAACCTGGGCACT-3'<br><br>Includes XhoI site (underlined) for cloning	27 bases	PCR reverse primer used for Preparation of *-3KCHRDL1-pGL4 Luc* construct
TCF3FW	5'-ATGAACCAGCCGCAGAGGATGG-3'	22 bases	PCR forward primer used for preparation of TCF3 expression construct in pCRII-TOPO vector
TCF3RV	5'-CGGAGGCATACCTTTCACAT-3'	20 bases	PCR reverse primer used for preparation of TCF3 expression construct in pCRII-TOPO vector
SP1 oligo	5'- ATTCGATCG*GGGCGG*GGCGAGC-3'<br>3'-TAAGCTAGC*CCCGCC*CCGCTCG-5'	22 base pairs	Non-specific competitor
CH2600TWI	5'-TATATACACAGG*CAAATG*AGTGCATATAAA-3'<br>3'-ATATATGTGTCC*GTTTAC*TCACGTATATTT-5'	30 base pairs	EMSA probe

CH2700EA	5'-TGGTGGTGGT*CACATG*GAATATA*CACATG*CGATAAAATT-3'<br>3'-ACCACCACCA*GTGTAC*TTATAT*GTGTAC*GCTATTTTAA-5'	38 base pairs	Specific competitor
CH2700EMAW	5'-TGGTGGTGGT*CGCACG*AATATA*CACATG*CGATAAAATT-3'<br>3'-ACCACCACCA*GCGTGC*TTATAT*GTGTAC*GCTATTTTAA-5'	38 base pairs	Competitor
CH2700EWAM	5'-TGGTGGTGGT*CACATG*AATATA*CGCACG*CGATAAAATT-3'<br>3'-ACCACCACCA*GTGTAC*TTATAT*GCGTGC*GCTATTTTAA-5'	38 base pairs	Competitor

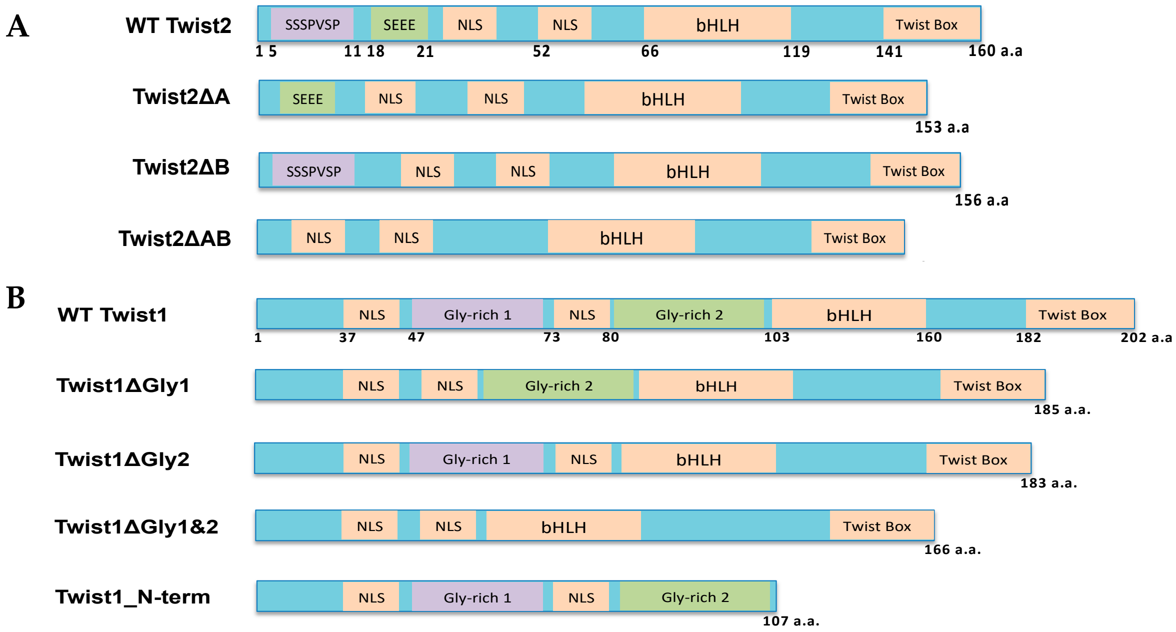

Supplementary Figure S1. Diagram of the TWIST2 and TWIST1 N-terminal mutant proteins produced by deletion mutagenesis. A. We generated mutant TWIST2 expression constructs where we removed two conserved sub-motifs [43], which were predicted to be associated with protein binding, the first sub-motif SSSPVSP (construct TWIST2ΔA), the second sub-motif SEEE (construct TWIST2ΔB), or both sub-motifs (construct TWIST2ΔAB). B. Diagram of TWIST1 glycine-rich deletion mutant proteins and the N-Terminal construct used for EMSAs, which were generated by gene synthesis. NLS = nuclear localization signal; bHLH = basic helix loop helix; TWIST box = protein domain which interacts with the Runx2 DNA binding domain to inhibit its function [9].

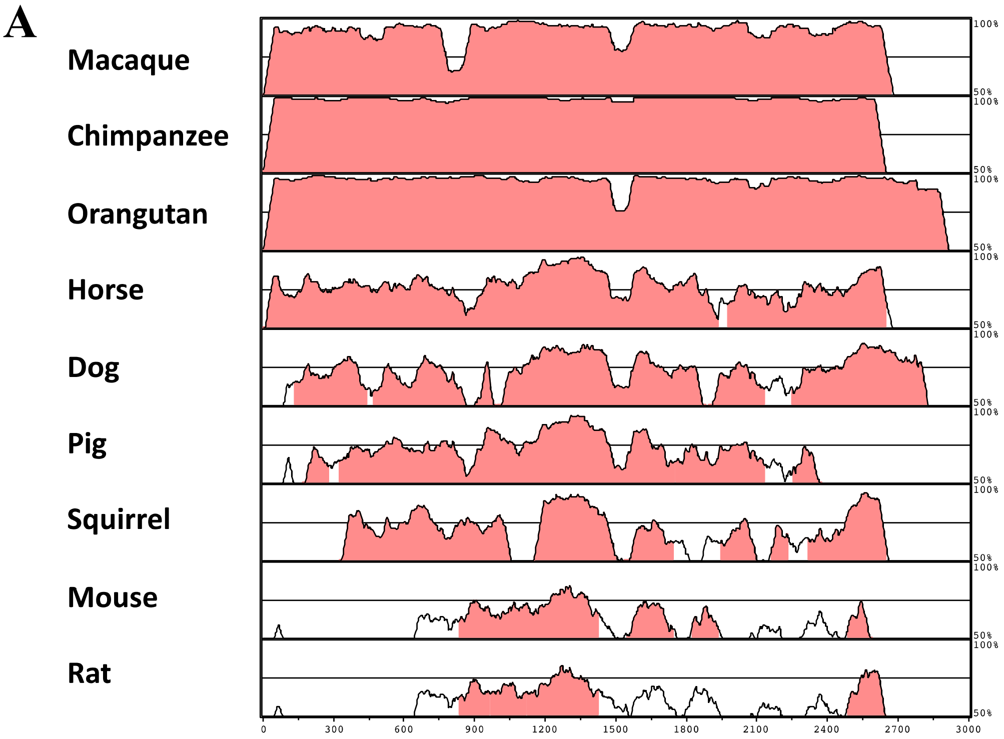
A
Macaque
Chimpanzee
Orangutan
Horse
Dog
Pig
Squirrel
Mouse
Rat
100%
50%
0
300
600
900
1200
1500
1800
2100
2400
2700
3000

B.

```
                                      -1239Twist
Human        000002058  AACTC-TAACACTT-------GAGACCTCAGGTAGTT----CGGTTGGTT  000002095
Macaque      000002144  AACTCTTAACACTT-------GAGACCTCAGGTAGTT----CGGTTGGTT  000002182
Chimpanzee   000002112  AACTCTTAACACTT-------GAGACCTCAGGTAGTT----CGGTTGGTT  000002150
Orangutan    000001739  AACTC-TAACGCTT-------GAGACCTCAGGTAGTT----CGGTTGGTT  000001776
Horse        000002139  AACAC-TGACACTT-------CAGGCCACAGACAGTTCA--CAGTTTGTT  000002178
Dog          000002151  GGATTCTGACACCG-------GAGGCCACAGACAGTTCAC--AGTTGGTC  000002191
Pig          000001808  AACTT-TAACACTT-------GAGGCTATACAGAGATAATTCAGTTTGTT  000001849
Squirrel     000001470  AACT---CACACTT-------GAAGCCACTGATAGTTC----AGTTTCTT  000001505
Mouse        000002160  --------GCATCTCATCAGGAAGGCTACTGATAGTTTATTTAATTTGTA  000002201
Rat          000002104  --------GCATCTCTTAAGAAAAGCTACTGATAACTTATTAAGTTTGTA  000002145
```

C.

```
                                                         -2661SREBP1 -2648SREBP1
Human        000000616  CTGTTTTATATTTTTACTGTGGTGGTGGTCACATGAATATACACATGCGA  000000665
Macaque      000000708  CTGTTCTATATTTTTACTGTGGTGGTGGTCACATGAATGTACACATGTGA  000000757
Chimpanzee   000000677  CTGTTCTATATTTTTACTGTGGTGGTGGTCACATGAATGTACACATGTGA  000000726
Orangutan    000000280  CTGTTTTATATTTTTACTGTGGTGGTGGTCACATGAATATACACATGTGA  000000329
Horse        000000722  CTGTTCAGTATCTTGATTGTGGCGCTGGTCACATGAACCCACACATGTGA  000000771
Dog          000000535  TTGTTCAGTATTCTGACTGTGGTGGTGGCCACATGAAGCAATACATGTGA  000000584
Pig          000000377  TTG---------TTGACTGTGGTGGTGATTGCATCAACCTACATATGTGA  000000417
Squirrel     000000096  CTGTTTTGTATCCTGACTGTGGTGGTGATCACCTGAACCTACCTATGTGG  000000145
Mouse        000000000  --------------------------------------------------  000000000
Rat          000000000  --------------------------------------------------  000000000

                                                        -2611Twist
Human        000000666  TAAAATTGCATAGAATTATATACACAGGCAAATGAGTGCATATAAAACTG  000000715
Macaque      000000758  TAAAATTGCATAGAATTATATACATAGGCAAATGACTGGATATAAAACTG  000000807
Chimpanzee   000000727  TAAAATTGCATAGAATTATATACATAGGCAAATGACTGGATATAAAACTG  000000776
Orangutan    000000330  TAAAATTGCATAGAATTATATACACAGGCAAATGAGTACATATAAAACTG  000000379
Horse        000000772  TAAAATTTCATAGAACTATACACATAGGCAAATGGGTGCACGTAAAAGTG  000000821
Dog          000000585  TAAAATTTCATATAACTATATACACAGGCAAATGGATGCATGCAAAACTG  000000634
Pig          000000418  TAAAATTTCCTAGAGCTATACGCATAGGCAGATGGGTGCGTATAAATG-A  000000466
Squirrel     000000146  TAAAATTGCGTAGAATTATACATGCAGGCAAATGAGTGCATGTAAAACTG  000000195
Mouse        000001075  -------------------------AGGCACA------------------  000001081
Rat          000001023  -------------------------AGGCACA------------------  000001029
```

Supplementary Figure S2. Bioinformatic analysis of the 5′ upstream region of the human *CHRDL1* gene to identify putative TF binding sites and sequence conservation A. mVISTA bioinformatic analysis of the upstream region (-3000 to +1) of the *CHRDL1* gene among selected mammals. Schematic representation of the global multiple sequence alignment of the different animals when compared to the human *CHRDL1* gene upstream sequence. Red areas indicate conservation based on mVISTA parameters. B) Multiple sequence alignment of the E-boxes found in probe #3 (from -1297 to -1148). C) Multiple sequence alignment of the E-boxes found in probe #5 (from -2697 to -2548). The region contained in probe #5 is the most conserved in most of the species considered in the mVISTA analysis. The -2661 site is conserved in human, macaque, chimpanzee, orangutan, horse, dog, and squirrel. The -2648 site is conserved in human, macaque, chimpanzee, orangutan, horse, and pig. Interestingly the -2611 TWIST site is conserved in 8 out of 10 species considered in this analysis.

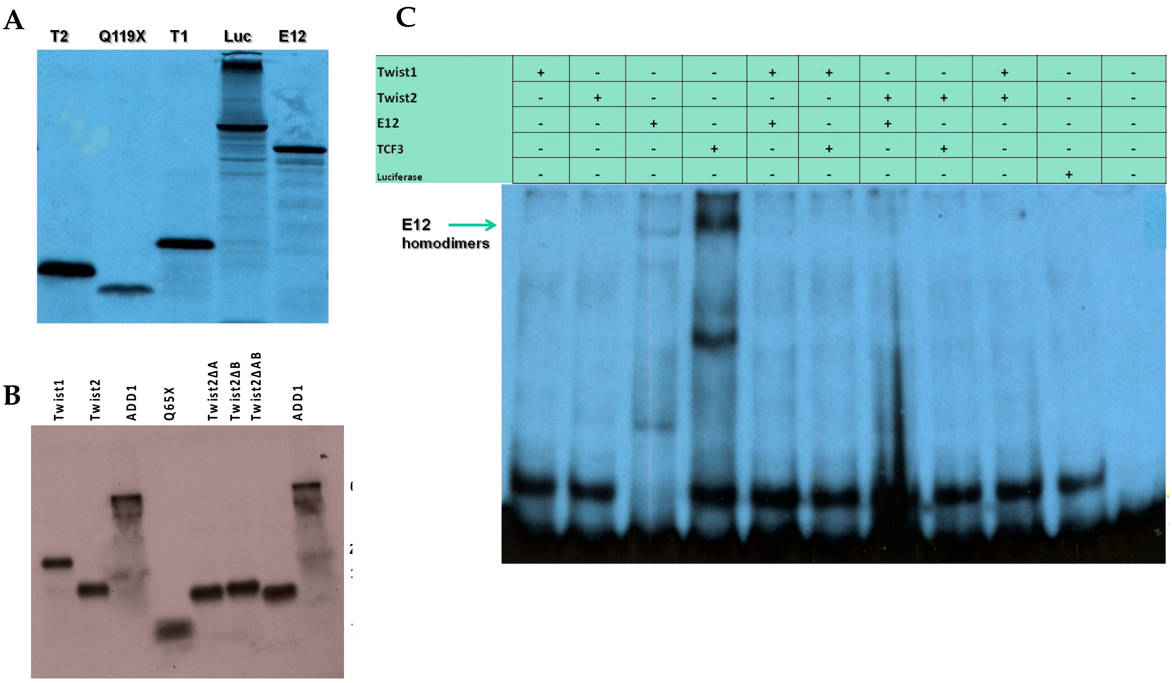

Supplementary Figure S3. SDS-PAGE of *in vitro* transcribed/translated proteins and Electrophoretic Mobility Shift Assay for probe #1 of the *CHRDL1* gene. A. This SDS-PAGE autoradiography represents an example of the typical results obtained for the proteins synthesized *in vitro* using the TnT Quick-Coupled Transcription/Translation kit (TnT) and labeled by incorporation of ^{35}S-Methionine to assess protein production. **B.** Western Blot of SDS -PAGE of TnT reactions of TWIST proteins and ADD1, detected with an anti-Myc antibody. Luciferase was used as a positive control for protein synthesis and as a negative control (mock) for EMSA binding reactions. In both A and B, similar amounts of protein were obtained for most proteins. Abbreviations used: T2=TWIST2; Q119X and Q65X= mutant forms of TWIST2 found in Setleis Syndrome patients, T1= TWIST1; Luc=Luciferase. **B.** EMSA carried out with *in vitro* synthesized proteins using probe #1. For this probe we detected binding of E12 and in the reaction with the TCF3 construct, which codes for E12 as well. The region in probe #1 contains a putative binding site for E12 at position -167 and a TWIST site at -194.

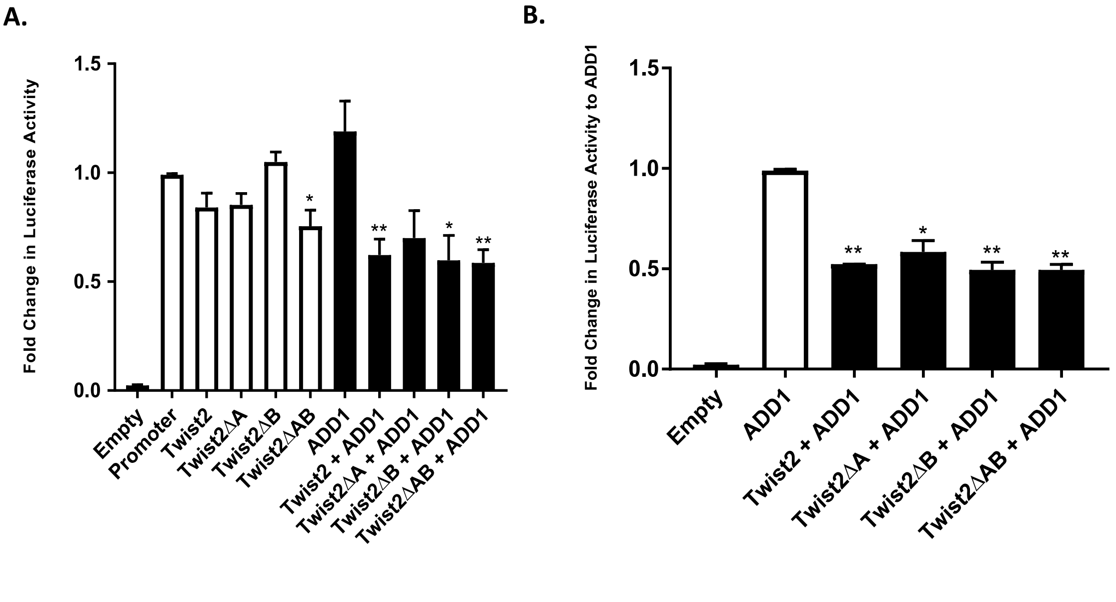

Supplementary Figure S4. Suppression of the transcriptional activity of ADD1/ SREBP1c by TWIST2 and its deletion mutant N-terminal forms in HeLa cells. Luciferase reporter gene assay using HeLa cells were used to assess the effect of the different proteins (TWIST2, TWIST2ΔA, TWIST2ΔB, TWIST2ΔAB and ADD1) on the *CHRDL1* gene upstream region. **A.** Comparison of the basal levels of reporter expression (promoter) signal to the expression signal obtained when transfected with the constructs observed in the figure. All comparisons were made relative to the basal promoter activity. **B.** The expression levels of the co-transfected reactions were compared to ADD1-dependent activation of the *CHRDL1* gene promoter (reporter expression activity was normalized against the expression of the reporter gene when transfected with ADD1). Transfection assays were performed in duplicate and repeated independently at least three times (N=3). Statistical analysis was done using one-way ANOVA followed by unpaired Student's T-test. Error bars represent standard error of the mean (SEM). *= $p<0.05$, **= $p<0.01$.
